# Time Trend in SARS-CoV-2 Seropositivity, Surveillance Detection- and Infection Fatality Ratio until Spring 2021 in the Tirschenreuth County—Results from a Population-Based Longitudinal Study in Germany

**DOI:** 10.3390/v14061168

**Published:** 2022-05-27

**Authors:** Sebastian Einhauser, David Peterhoff, Stephanie Beileke, Felix Günther, Hans-Helmut Niller, Philipp Steininger, Antje Knöll, Klaus Korn, Melanie Berr, Anja Schütz, Simon Wiegrebe, Klaus J. Stark, André Gessner, Ralph Burkhardt, Michael Kabesch, Holger Schedl, Helmut Küchenhoff, Annette B. Pfahlberg, Iris M. Heid, Olaf Gefeller, Klaus Überla, Ralf Wagner

**Affiliations:** 1 Institute of Medical Microbiology and Hygiene, Molecular Microbiology (Virology), University of Regensburg, Franz-Josef-Strauß-Allee 11, 93053 Regensburg, Germany; sebastian.einhauser@klinik.uni-regensburg.de (S.E.); david.peterhoff@klinik.uni-regensburg.de (D.P.); hans-helmut.niller@klinik.uni-regensburg.de (H.-H.N.); melanie.berr@klinik.uni-regensburg.de (M.B.); anja.schuetz@klinik.uni-regensburg.de (A.S.); Andre.Gessner@klinik.uni-regensburg.de (A.G.); 2 Institute of Clinical Microbiology and Hygiene, University Hospital Regensburg, Franz-Josef-Strauß-Allee 11, 93053 Regensburg, Germany; 3 Institute of Clinical and Molecular Virology, University Hospital Erlangen, Friedrich-Alexander Universität Erlangen-Nürnberg, Schlossgarten 4, 91054 Erlangen, Germany; stephanie.beileke@uk-erlangen.de (S.B.); philipp.steininger@uk-erlangen.de (P.S.); antje.knoell@uk-erlangen.de (A.K.); klaus.korn@uk-erlangen.de (K.K.); 4 Department of Mathematics, Stockholm University, Kräftriket 6, 106 91 Stockholm, Sweden; felix.gunther@math.su.se; 5 Department of Genetic Epidemiology, University of Regensburg, Franz-Josef-Strauß-Allee 11, 93053 Regensburg, Germany; simon.wiegrebe@ur.de (S.W.); klaus.stark@ur.de (K.J.S.); iris.heid@ur.de (I.M.H.); 6 Institute of Clinical Chemistry and Laboratory Medicine, University Hospital Regensburg, Franz-Josef-Strauß-Allee 11, 93053 Regensburg, Germany; ralph.burkhardt@klinik.uni-regensburg.de; 7 University Children’s Hospital Regensburg (KUNO) at the Hospital St. Hedwig of the Order of St. John, University of Regensburg, Steinmetzstraße 1-3, 93049 Regensburg, Germany; michael.kabesch@barmherzige-regensburg.de; 8 Bayerisches Rotes Kreuz, Kreisverband Tirschenreuth, Egerstraße 21, 95643 Tirschenreuth, Germany; schedl@kvtirschenreuth.brk.de; 9 Statistical Consulting Unit StaBLab, Department of Statistics, LMU Munich, Geschwister-Scholl-Platz 1, 80539 Munich, Germany; kuechenhoff@stat.uni-muenchen.de; 10 Department of Medical Informatics, Biometry and Epidemiology, Friedrich-Alexander Universität Erlangen-Nürnberg (FAU), Waldstr. 6, 91054 Erlangen, Germany; annette.pfahlberg@fau.de (A.B.P.); olaf.gefeller@fau.de (O.G.)

**Keywords:** SARS-CoV-2, seroprevalence, infection fatality ratio, case fatality ratio, surveillance detection ratio, senior care homes, elderly, vaccination, population-based, longitudinal

## Abstract

Herein, we provide results from a prospective population-based longitudinal follow-up (FU) SARS-CoV-2 serosurveillance study in Tirschenreuth, the county which was hit hardest in Germany in spring 2020 and early 2021. Of 4203 individuals aged 14 years or older enrolled at baseline (BL, June 2020), 3546 participated at FU1 (November 2020) and 3391 at FU2 (April 2021). Key metrics comprising standardized seroprevalence, surveillance detection ratio (SDR), infection fatality ratio (IFR) and success of the vaccination campaign were derived using the Roche N- and S-Elecsys anti-SARS-CoV-2 test together with a self-administered questionnaire. N-seropositivity at BL was 9.2% (1st wave). While we observed a low new seropositivity between BL and FU1 (0.9%), the combined 2nd and 3rd wave accounted for 6.1% new N-seropositives between FU1 and FU2 (ever seropositives at FU2: 15.4%). The SDR decreased from 5.4 (BL) to 1.1 (FU2) highlighting the success of massively increased testing in the population. The IFR based on a combination of serology and registration data resulted in 3.3% between November 2020 and April 2021 compared to 2.3% until June 2020. Although IFRs were consistently higher at FU2 compared to BL across age-groups, highest among individuals aged 70+ (18.3% versus 10.7%, respectively), observed differences were within statistical uncertainty bounds. While municipalities with senior care homes showed a higher IFR at BL (3.0% with senior care home vs. 0.7% w/o), this effect diminished at FU2 (3.4% vs. 2.9%). In April 2021 (FU2), vaccination rate in the elderly was high (>77.4%, age-group 80+).

## 1. Introduction

Despite a slight relief in new SARS-CoV-2 infections in summer 2020 following the 1st wave and several highly effective vaccines becoming available [1,2,3], most European countries including Germany were facing new waves of infections from autumn 2020 till now and emerging variants of concern, namely alpha [4], delta [5], and omicron [6,7].

Early during the SARS-CoV-2 pandemic, multiple population-based cross-sectional seroprevalence studies have been initiated across the globe in order to calculate the proportion of undetected infections and the infection fatality ratio (IFR) [8]. The ratio of actual infections to the number of registered infections is a measure of the surveillance success (surveillance detection ratio, SDR) and differs between populations and age groups, e.g., due to the intensity of testing and symptom severity [9]. The IFR is a hallmark of the severity of the pandemic and one of the prime reasons for containment measures. IFR has been shown to depend substantially on age [8] and the extent that the particularly vulnerable part of the population, like senior care home residents, were included [9]. An upper bound for the IFR is the case-fatality-ratio (CFR) derived from the proportion of registered COVID-19 deaths to registered infected. As long as registered infected underreport the true number of infected, the CFR is an overestimate of the IFR. 

SDR and IFR are highly relevant as key metrics to guide and judge political action such as testing strategy, containment measures, and vaccination campaigns. To understand changes over time, longitudinal studies investigating a defined group of individuals (cohort) over time are required to adequately address the longitudinal dynamic in SDR and IFR. 

However, systematic longitudinal seroprevalence studies reporting interval-related incidences of new infections, SDR, and IFR are scarce. Most studies assessing population-based seropositivity over time repeat cross-sectional analyses at consecutive time points in different panels, focus on the increase in total seroprevalence and rarely report IFRs [10,11,12,13,14]. For Germany, the Robert-Koch Institute (RKI, CDC equivalent) reports 26 population-based cross-sectional seroprevalence studies in Germany in mostly low-incidence populations [15,16,17], selected hotspots [18] or distinct sub-groups [19,20,21,22]. On top there is the recently pulished, federal state wide SaarCoPS study [23] which thoroughly assessed key metrics during the first wave. Only two studies included serial assessments over time. The KoCo19 study [16] assessed households longitudinally between November 2020 until October 2021. MuSPAD, the “Multilocal and Serial Prevalence Study of Antibodies against SARS-2 Coronavirus” provided estimates of seropositivity, SDR, and IFR across different regions for up to 2 time points, but for different panels of individuals, which hampers the comparison over time (serial study, between July 2020 and May 2021) [13]. While SaarCoPS rather focused on the influence of various tests on key metrics than on different time points [23]. In addition to the above-mentioned metrics, population-based longitudinal studies can also provide important insights with regard to infection- and vaccine-induced immunity over time [24]. 

Results of longitudinal population-based studies are impacted e.g., by triggering events or regional differences in the infection intensity. KoCo19, SaarCoPS and also MuSPAD for example describe serology-based metrics in populations with low incidence during the first wave in spring 2020, and so far only MuSPAD has computed SDRs and IFRs for multiple time points capturing also the rising infections in winter 2020/21. 

We thus set out to investigate the time trend in SARS-CoV-2 seropositivity, seroconversion from positive to negative, SDR and IFR over time in the Tirschenreuth county in Northeastern Bavaria, Germany. Tirschenreuth was one of the hardest hit counties in Germany during the 1st wave in spring 2020 [25,26] and in early 2021 (combined 2nd and 3rd wave caused by the SARS-CoV-2 wild type variant D416G and the alpha variant). For this, we conducted two follow-up investigations in November 2020 and April 2021 (FU1, FU2, respectively) in our established seroprevalence cohort study with baseline assessment in June 2020 [9]. We invited >4100 study participants aged 14 to 102 years for these two follow-up blood draws in the same fashion as for baseline and yielded measured N- directed SARS-CoV-2 antibodies of up to three time points. We also measured S-directed antibodies for the same individuals and time points to compare S-test with N-test derived seroposivitiy and seronegativity. Since vaccination started in Germany at 27 December 2020, our S-test measurements provide additional insights into vaccination rates at FU2.

## 2. Materials and Methods

### 2.1. Cohort Design, Inclusion Criteria, and Study Program

The cohort at baseline (BL) was described in detail in Wagner et al. 2021 [9]. In brief, 4203 randomly selected inhabitants of Tirschenreuth aged 14 years or older participated in the baseline survey of the TiKoCo study (response rate: 64.3%). Study participants came to the study center or requested a visit at home between 28th June and 13th July 2020.

For the longitudinal follow-up (FU), 4173 of the 4203 BL participants who had agreed to be re-contacted were re-invited for a second (16th to 27th November 2020; FU1) and third blood sampling (19th and 30th April 2021; FU2) in the same fashion as for BL assessment [9]: Handicapped or otherwise immobile participants (*n* = 112 and 79 at FU1 and FU2, respectively) were visited at home; invited individuals with flu-like symptoms were asked to stay at home and use the installed hot-line to arrange an appointment for a home visit. At BL, FU1, and FU2, participants were asked to provide blood (5.7 mL) and to fill out a questionnaire (see below). 

The TiKoCo study was approved by the Ethics Committee of the University of Regensburg, Germany (vote 20-1867-101) and adopted by the Ethics Committee of the University of Erlangen (vote 248_20 Bc). The study complies with the 1964 Helsinki declaration and its later amendments. All participants provided written informed consent.

### 2.2. Data on Registered COVID-19 Related Deaths, Registered Infected, and Tirschenreuth County Inhabitants

From local health authorities, we obtained sex-, age- and municipality-specific numbers of COVID-19 related deaths and registered infections. The number of inhabitants of the Tirschenreuth county, by sex, age-groups, and municipalities, were obtained by the municipal administration (as of December 2019). For the Tirschenreuth county population, we assumed a steady state, i.e., a similar number of inhabitants and sex- and age-group distributions across years. We also derived the number of Tirschenreuth county inhabitants living in a care home as well as respective COVID-19 related deaths and registered cases by the county administration. 

### 2.3. Observation Periods

Our longitudinal seroprevalence study consisted of three observation periods: (i) from pre-pandemic until BL blood draw; (ii) between BL and FU1 blood draw; (iii) between FU1 and FU2 blood draw. 

To define precise observation periods for registered COVID-19 cases and COVID-19 deaths, the time interval from first symptoms to seroconversion was assumed to be 12 days [27], from first symptom to registration as COVID-19 case with the RKI 8 days [28], and from first symptoms to COVID-19 associated death 16 days [29]. The cut-off date for registered COVID-19 cases and COVID-19 associated deaths were therefore defined as the fourth day prior to the median day of sampling and the fourth day after the median day of sampling, respectively. 

This resulted in the following observation periods for registered COVID-19 cases: observation period 1 (until BL): 1 February to 4 July 2020; observation period 2 (BL to FU1): 5 July to 18 November 2020; observation period 3 (FU1 to FU2): 19 November to 21st April 2021. The observation periods for COVID-19 associated deaths were 1 February to 12 July 2020, 13 July to 26 November 2020, and 27 November to 29 April 2021, respectively.

### 2.4. Assessment of Educational Status, Comorbidities, Self-Reported Previous Infections, and Vaccination Status

A questionnaire was designed and administered at BL as previously described [9] and analogously administered for FU1 and FU2. In brief, the self-administered questionnaire was sent out with the invitation and collected at the study center (or at home), with the possibility of personal counseling by trained staff in case of questions. At BL, participants were asked if they had been tested for SARS-CoV2, whether the test was positive, which current diagnoses of chronic diseases they had, which school and further education they had undergone, and whether they were living in a care home. At FU1 and FU2, participants were further asked with regard to testing, if tested positive since last visit, and with regard to existing chronic disease diagnoses. At FU2, individuals were also asked about the number of received vaccinations, including respective date(s) and type(s). 

### 2.5. Blood Sampling, Transport and Antibody Measurements

Blood sampling and transport of samples was performed as previously described [9]. In brief, after blood drawal by qualified study personnel into a barcoded serum monovette (Sarstedt AG Co.KG, Nümbrecht, Germany), the serum sample was processed on the same day. To assess SARS-CoV-2 antibodies, we used an Elecsys Anti-SARS-CoV-2 N test (Roche Diagnostics GmbH, Penzberg, Germany) detecting nucleoprotein-(N)-directed complete Ig as well as the Elecsys Anti-SARS-CoV-2 Spike test (Roche Diagnostics GmbH, Penzberg, Germany) detecting Spike-protein (receptor-binding-domain; RBD) directed complete Ig (referred to as “N-test” and “S-test”, respectively). Both tests were operated on the Cobas pro e 801 module. Sensitivities and specificities provided by the manufacturers in the instructions for use are 99.5% and 99.8%, respectively, for the ELECSYS anti-SARS-CoV-2 N (Roche Diagnostics) or 98.8% and 99.98% for the ELECSYS anti-SARS-CoV-2 S (Roche Diagnostics). We decided to focus our follow-up analyses on these two Roche, ELECSYS tests because: in the BL analysis, we observed that ELECSYS N results were most in line with the results from latent class modeling using information from three antibody tests, including the YHLO and our in-house S_RBD_-ELISA [9,30]; ELECSYS S test provided excellent concordance with a latent class model and neutralization as published earlier [31]. Finally, the use of these two tests also enabled the discrimination of serostatus resulting from infection versus vaccination. Test results were also reported to study participants to incentivise participation in the study.

### 2.6. Statistical Analysis

**Figure 1:**
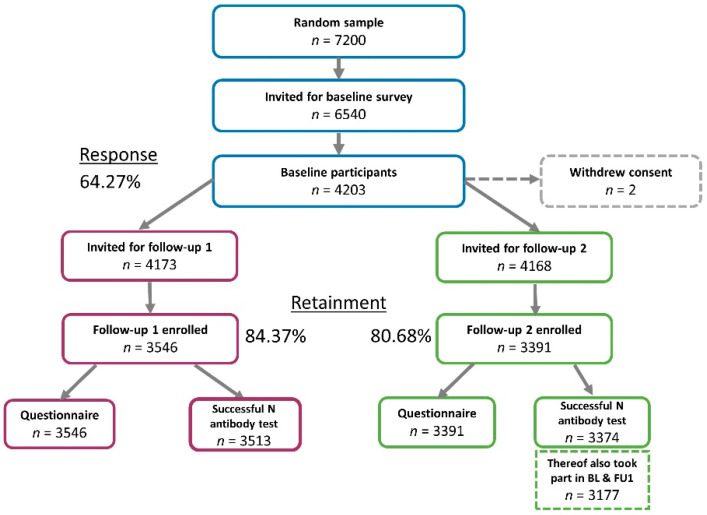
Summary of the TiCoKo longitudinal study design—follow-up 1 and follow-up 2. Shown are the numbers of invited individuals (14 years and older), follow-up participants, information via questionnaire, successful blood-draws and available N-test measurement for follow-up 1 and 2, respectively.

From these longitudinal analyses, we excluded 17 individuals without successful N-test measurement at BL and 33 and 17 individuals without successful N-test measurements at FU1 or FU2, respectively (Figure 1). We compared BL characteristics of FU1 and FU2 participants with those of drop-outs, using a *t*-test or Chi-square test (judged at 5% significance level). 

For each of the three observation periods (until BL, BL to FU1, FU1 to FU2), we computed the % new seropositives in the study sample using the anti-SARS-CoV-2 N-test to estimate the probability of a participant to be seropositive at the end of the observation period given that the person was seronegative at the end of the previous observation period (i.e., at risk for seroconversion into positive): (i) % (new) seropositives at BL as the number of seropositives at BL divided by all participants with N-measurement at BL, (ii) % new seropositives at FU1 (compared to BL) as the number of individuals seropositive at FU1 AND seronegative at BL divided by the number of seronegatives at BL, and (iii) % new seropositives at FU2 (compared to FU1) as the number of individuals seropositive at FU2 AND seronegative at FU1 divided by the number of seronegatives at FU1 (restricted to those with N-test available at FU2). We did this overall, by sex and by age-groups. 

Given the time points of BL, FU1 and FU2 at June 2020, November 2020, and April 2021, these % new seropositives reflect the % infected during the 1st wave until July 2020, during the low incidence period in summer 2020, and during the 2nd/3rd wave of autumn/winter 2020/21, respectively. Of note, the total seropositivity at FU2 does not reflect the % infected until FU2, if individuals seroconverted from positive to negative.

The total % seropositives at a specific time point, *t*, can also be derived in a sequential approach: let *t* = 1, 2, 3 for BL, FU1 and FU2, respectively; *t* = 0 reflects the time before the pandemic. The probability (P) for a person to be seropositive at time *t* can be written, according to Bayes’ theorem as:

P(seropos. at t) = P(seropos. at t|seroneg. at t − 1) * P(seroneg. at t − 1) + P(seropos. at t|seropos. at t − 1) * P(seropos at t − 1) (1)

with P(seropos at t = 0) = 0. The probability *P(seropos. at t|seroneg. at t − 1)* can be estimated from serology data as the % new seropositives in the time period between *t* *−* 1 and *t* (assuming the number of seroconverters from positive to negative within the time period *t* ‒ 1 to *t* to be few and thus negligible). Accordingly, 1 *− P(seropos. at t|seropos. at t − 1)* is reflected by the % new seronegatives (i.e., the seroconverters from positive to negative). Thus, the sequential approach to derive the % total seropositivity can be derived as

% total seropos. (at t) = % new seropos (t − 1 to t) * (100% − %total seropos(at t − 1)) + (100% − % new seroneg(t − 1 to t))* %total seropos (at t − 1) (2)

The *% ever seropos. until t* includes the individuals seroconverting from positive to negative and can be approximated by

% ever seropos. (at t) = % new seropos (t − 1 to t) * (100% − %total seropos(at t − 1)) + %total seropos (at t − 1) (3)

The *% ever seropos. until FU1 or FU2* are thus an estimate of the % infected until FU1 or FU2, respectively that can’t be estimated based on the cross-sectional data of FU1 or FU2 alone. In contrast, the *% total seropositives (at t)* can in principle be estimated based on cross-sectional data as the fraction of seropositives at the respective time point. However, the sequential estimation approach has a considerable advantage: usually, study participants in a longitudinal seroprevalence study are informed of their antibody status after each study time point. However, knowledge of the test result may have an effect on future willingness to participate at follow-up assessments. For example, if a study participant only learns of a previous infection through a positive antibody test and wishes to have this result confirmed in a later assessment. In this case, the cross-sectional estimate of *% total seropositives (at t)* can be substantially biased. The sequential approach can still yield unbiased estimates when participation at follow-up assessments is associated with antibody status at previous time points.

### 2.7. Standardization

For each of the three observation periods, we standardized the observed (crude) % new seropositive to the Tirschenreuth population as described previously for baseline [9]. In brief, we standardized crude seropositivity by age-group-, sex-, and municipality-specific weights according to Tirschenreuth county administration data. We also accounted for the COBAS test’s sensitivity and specificity by the approach of Sempos and Tian [32]. 

From the standardized % new seropositives at BL, FU1, and FU2, we derived the estimated number of infected individuals in the Tirschenreuth population for the respective observation periods (overall, by sex, and by age-groups). However, a specific challenge is the high COVID-19 related mortality particularly among older individuals: the individuals having died from COVID-19 were infected, but not covered by this—or any—seroprevalence study. We thus added the number of registered COVID-related deaths in the Tirschenreuth county for the respective observation period to our derived expected number of infected. Dividing this expected number of infected accounting for deaths by the number of inhabitants (overall, by sex, by age-groups), we obtained estimates for infection incidence during the three observation periods. 

For each of the three observation periods, we derived the SDR as the expected number of infected accounting for deaths divided by the number of registered infected in the Tirschenreuth county. The SDR is a measure with a lower bound of 1.0. When the number of expected infected in the county based on serology was lower than the number of registered cases, the SDR was set to 1.0. We did this overall, by sex, and by age-groups. 

The CFR compares registered COVID-19 related deaths with the registered infected (“cases”) and over-estimates fatality when testing strategies are incomplete and not all infected are registered. The IFR is the number of deaths divided by the true number of infected in the population. Standardized seroprevalence estimates from population-based studies are considered state-of-the-art to determine the number of infected in populations. For each of the three observation periods, we derived the IFR (overall, by sex, and by age-groups) as the registered number of SARS-CoV-2 related deaths in the Tirschenreuth county divided (i) by the number of infected calculated based on our serology data, or (ii) by the number of registered infected if this number was higher than the number resulting from serology (the latter is, in fact, the CFR). 

Despite the offer of a home visit by the study team, participation rate of senior care home residents was low rendering the calculation of estimates for this group impossible. As an approximation, we approached a possible impact of senior care homes on the overall IFR by dividing the municipalities of the county into two subgroups: municipalities with at least one senior care home versus municipalities without any senior care home (as described previously for the BL analysis [9]).

### 2.8. Confidence and Credibility Intervals

All 95%-CIs for standardized seropositivity were computed using Wilson’s method for binomial proportions assuming the weights as fixed constants. For the standardized IFRs and SDRs, we computed Bayesian credibility intervals accounting also for variation in the number of reported cases or deaths (following the reasoning of and using a similar method as Streeck et al. [18]). For the IFR, we first obtained 100,000 random draws from two Beta-distributions with parameters $a_{1}=\#\left(\mathrm{reported}\;\mathrm{deaths}\right)+1$ and $b_{1}=\;\#\left(\mathrm{at}\;\mathrm{risk}\;\mathrm{in}\;\mathrm{population}\right)-\#\left(\mathrm{reported}\;\mathrm{deaths}\right)+1$ and $a_{2}=\;\#\left(\mathrm{est}.\;\mathrm{new}\;\mathrm{seropos}.\;\mathrm{in}\;\mathrm{study}\right)+1$ and $b_{2}=\;\#\left(\mathrm{at}\;\mathrm{risk}\;\mathrm{in}\;\mathrm{study}\right)-\#\left(\mathrm{est}.\;\mathrm{new}\;\mathrm{seropos}.\;\mathrm{in}\;\mathrm{study}\right)+1$, where #(est. new seropos. in study) is the expected number of new seropositive cases in our study (i.e., after standardization and accounting for COVID-19 related deaths and for imperfect sensitivity/specificity of the N-test). These Beta-distributions correspond to posterior distributions of the binomial proportions for the number of deaths among the population and the number of seropositives among the study participants, respectively. We divide samples from these posteriors and calculate the 2.5% and 97.5% quantiles of the ratio to derive approximate 95% credible intervals for the IFR. When using registered cases instead of the expected number of new sero-positives in the population as denominator (i.e., the CFR), $a_{2}$ and $b_{2}$ are replaced by $a_{2}=\;\#\left(\mathrm{reg}.\;\mathrm{cases}\;\mathrm{in}\;\mathrm{population}\right)+1$ and $b_{2}=\;\#\left(\mathrm{at}\;\mathrm{risk}\;\mathrm{in}\;\mathrm{population}\right)-\#\left(\mathrm{reg}.\;\mathrm{cases}\;\mathrm{in}\;\mathrm{population}\right)+1$. Credible intervals for SDRs are constructed in a similar fashion by dividing samples from the Beta-posterior of the binomial fraction of new seropositives in our study and new registered cases in the population. We refrain from providing credible intervals for the SDRs when there are fewer expected new infections in the population (based on serology) than registered infections.

### 2.9. Vaccination

To obtain the proportion of individuals that were vaccinated in the study sample, we considered the questionnaire-based information on vaccination status: an individual was considered fully vaccinated when having received two doses of Comirnaty at least 14 days prior to blood draw. To obtain the antibody profile in vaccinated and/or infected, we also derived the individuals’ status of being seropositive for S, but not for N (i.e., seroconversion due to vaccination, not due to infection) and the individual’s status of being seropositive for S and N (infected or infected and vaccinated) at FU2, divided this by the number of individuals with S-measurement at FU2 and standardized this proportion to the Tirschenreuth county population as described above.

## 3. Results

### 3.1. Participant Characteristics and Dropout Analysis

**Figure 2:**
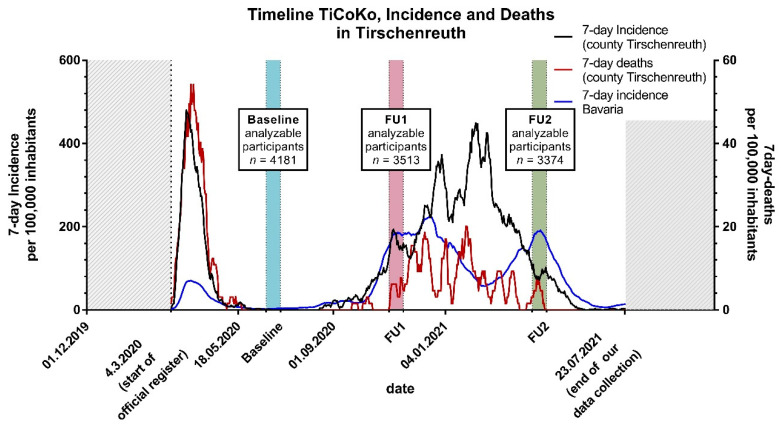
Overview of the time of assessments in the context of Tirschenreuth county SARS-CoV-2 infection waves. Shown are, over time, the 7 day registered infection incidence, the 7-day registered COVID-19 related death incidence per 100,000 of Tirschenreuth county inhabitants and the time of study assessments. Also shown are the numbers of enrolled participants and the number of analyzable participants, i.e., with successful N test measurements at BL, at FU1 and BL, and at FU2 and BL, respectively. N-test measurements for all three time point were available for *n* = 3177.

Among the 4203 participants enrolled at BL, 3546 participated in FU1 (November 2020; 84.1%) and 3391 in FU2 (April 2021; 80.4%). Overall 3196 participants took part in all BL, FU1 and FU2, resulting in a full participation ratio of 76.0%. (Figure 1 and Figure 2). 

Among the 4203 enrolled participants at BL, 4181 had N-test measurements available at BL. Among these, 4181, 3513 and 3374 participants had N-test measurements available at BL, FU1, or FU2, respectively, and comprised the analyzed individuals for each of the three observation periods. For 3177 participants, N-test measurements were available for all three time points. Analyzable participants at BL were 48% men and aged 14 to 102 years with a median age of 52.0 years (Table 1). 

When comparing the characteristics of FU1 and FU2 participants with respective dropouts, we found significant difference regarding age and sex, but not with regard to reported comorbidity or education (*p* < 0.05) (Table 1 and Supplementary Table S1). A significant difference was also found with regard to seropositivity at BL, which was consistently higher in those returning at FU1 and FU2 (Table 1 and Supplementary Table S1), reasoning individuals seropositive at BL were more interested in the follow-up than individuals seronegative at BL. Total N-seropositivity at FU1 or FU2 can thus be biased by the confounding effect of differential response with regard to seropositivity at BL. Estimation of new seropositivity at FU1 among those seronegative at BL and at FU2 among those seronegative at FU1 is not biased by this differential response. This further supported our approach to derive total % of ever seropositives and the % seropositives at FU1 or FU2 in a step-wise approach (see Section 2.2). 

### 3.2. Crude N-Antibody Seropositivity over Time

For the three observation periods, we analyzed the 4181, 3513, and 3177 participants with N-test measurements available for BL, for both FU1 and BL, and for all three visits at FU2, FU1 and BL, respectively. We found % (new) seropositives of 8.95%, 0.66%, and 5.80% for the three observation periods, respectively (Table 2), with little differences by sex, but larger differences by age-groups (Supplementary Table S2). This reflects the low spread of SARS-CoV-2 during summer and autumn 2020 and the high infection occurrence in the combined 2nd and 3rd wave during winter and spring 2021. The % ever seropositives in our cohort was 9.55% at FU1 and 14.80% at FU2. 

Among the 351 individuals positive at BL analyzable also at FU1 and FU2, 13 were seronegative at FU1 (3.70%) and 15 at FU2 (4.30%). Thus, >5 months after infection (i.e., the time between BL and FU1 as well as between FU1 and FU2), approximately 4% were seroconverters from N-antibody positive to negative (Table 2). Computing the % total seropositives by the sequential approach (Methods) resulted in 9.22% at FU1 and 14.09% at FU2 (8.95% at BL). 

Of note, the total N-antibody positivity estimated directly from antibodies at FU1 and FU2 rather than estimated by the sequential approach was 10.22% (FU1) and 15.68% (FU2), respectively. This is higher then than the 9.22% or 14.09% from the sequential approach as a result of the above noted differential response with regard to seropositivity at BL (Table 2).

### 3.3. Serology vs. Positive PCR (Self-Reported and Confirmed by Health Authorities) across the Observation Periods

We compared our serological results (anti-N serostatus) with a participants’ self-reports of positive SARS-CoV-2 test confirmed by health authorities (i.e., based on positive PCR tests). 

Until BL, 66 positive tests among the study participants were recorded by Tirschenreuth health authorities, of which 6.06% (*n* = 4) couldn’t be confirmed by serology. Between BL and FU1, those numbers increased to 31.58% (*n* = 6) antibody negative tests among 19 registered PCR positives, settling back to 5.23% (*n* = 8 of 161) unconfirmable results between FU1 to FU2. Though these inconsistent serostatus observations (especially at FU1) seemed to be substantial at first, they are in line with a reduced positive predictive value, even for highly specific diagnostic tests, in times of large testing activity and low disease prevalence. When focusing on individuals with a “false-positive” PCR test (individuals with positive PCR tests but negative antigen test) among all tested persons (i.e., self-reported PCR-tested in the respective observation period), we found such inconsistent results in 0.80, 0.56, and 0.51% of all tested for BL, BL to FU1, and FU1 to FU2, respectively. Such a joint probability of positive PCR-test and a negative antibody test, P(non-infected., PCR-pos.) = P(PCR-pos.|non-infected) * P(non-infected) = (1-specificity) * P(non-infected), is in line with the high published specificity [33] of the SARS-CoV-2 RT-qPCR of 99.5% and low infection occurrence. A part of the inconsistent observations can also be due to false-negative N-serology.

In conclusion, the overall 238 individuals with positive test until FU2 (reported via questionnaire and confirmed by local health authority), the serological test was positive at FU2 for *n* = 220 (92.44%), but negative for 18 (7.56%). The vast majority of these can be attributed to loss of antibodies over time or a false-positive PCR test, emphasized by increased impact in times of high testing frequency combined with low incidences (Table 3). 

### 3.4. Dynamics of Standardized N-Antibody Seropositivity across the Observation Periods

**Figure 3:**
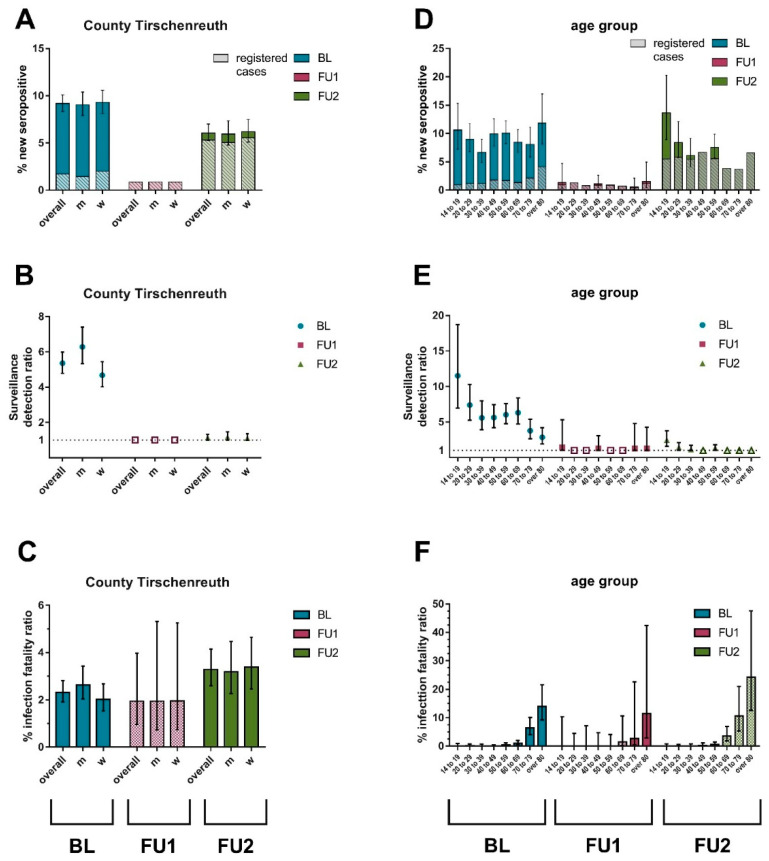
Standardized (N-based) new seropositivity, surveillance detection ratio (SDR), and infection fatality ratio (IFR) for Baseline (BL), Follow up 1 (FU1) and Follow up 2 (FU2). Shown are new seropositivity (based on N-antibodies) (%) and new registered case incidence (%), the surveillance detection ratio (SDR) and infection fatality ratio (IFR) (%) in the county population overall (**A**–**C**) and by age-groups (**D**–**F**). Non-solid coloring of symbols or bars (**B**,**C**,**E**,**F**) represents derived estimates from registered cases rather than serology, when registered cases exceeded the number of expected infections in the county based on serology. Error bars represent 95% confidence intervals (95%-CI, **A**,**D**) or 95% credibility intervals (**B**,**C**,**E**,**F**), respectively.

Based on the crude % new seropositives for each of the three observation periods, we accounted for antibody test misclassification, standardized to the Tirschenreuth county population aged ≥ 14 years, and added the (unobservable) deaths, in order to approximate the proportion of the Tirschenreuth county individuals that were infected within the respective periods. We refer to this as (standardized) % new seropositives in each of the three observation periods, which estimates the Tirschenreuth county (aged ≥ 14 years) cumulative incidence of infection of the respective time period. We found the highest proportion of (new) seropositives at BL (9.18%; 95%-CI 8.34–10.09; before June 2020), which captured the 1st SARS-CoV-2 wave as reported previously. We found only minor new N-seropositives (0.87%) between BL and FU1, which reflects the low-incidence period during summer 2020. Between FU1 and FU2, which captures the 2nd and 3rd wave, the new seropositivity was 6.06% (95%-CI 5.24–7.00) (Figure 3A, Supplementary Tables S3 and S4).

There were only little differences by sex, but age-stratified analysis revealed different patterns for different observation periods. While the seropositivity at BL was comparable across age groups, the new seropositivity between FU1 and FU2 was lower among the older aged population compared to the younger (Figure 3A,D; Supplementary Tables S3 and S4).

The % ever seropositives at the respective observation periods at BL, FU1 and FU2 was 9.18% [95%-CI 8.34–10.09], 9.97% [95%-CI 9.03–11.01] and 15.43% [95%-CI 14.25–16.69], respectively (Supplementary Figure S1 and Supplementary Table S5).

We also calculated % new seropositivity for each of the three observation periods as described above for each of the 26 municipalities in Tirschenreuth. At BL, municipality-specific seropositivity ranged from 1% [95%-CI 0.18–5.45] to 22.62% [95%-CI 14.49–33.52]. At FU1 compared to BL (July to November 2020), new seropositivity was negligible. At FU2 compared to FU1 (November 2020 to April 2021), new seropositivity ranged from 3.31% [based on registered cases] to 15.95% [95%-CI 14.49–33.52] (Supplementary Table S6). The total proportion of individuals infected until FU2 (% ever seropositive) ranged from 8.17% [95%-CI 3.69–17.12] to 34.07% [95%-CI 22.96–47.27] depending on the respective municipality (Supplementary Table S5).

### 3.5. Development of SDR and IFR across the Observation Periods

The German testing strategy at the beginning of the pandemic was limited in terms of test availability and logistics and therefore focused mainly on identifying symptomatic infections and contact tracing. This regime was reflected in our BL results, yielding an overall SDR of 5.35 (95%-CI 4.78–5.99), with higher SDR in the younger population (e.g., 13.64, 95%-CI 8.92–20.30, for 14 to 19-year-old) and lower SDR in the elderly (e.g., 2.84, 95%-CI 1.90–4.20, for the 80+) (Figure 3B; Supplementary Table S3). 

Our calculated SDRs for FU1 or FU2 were—for some age groups—below unity. In these instances, the SDR was set to 1.0. For the observation period between BL and FU1, we observed an SDR of 1.44 [95%-CI 1–5.31] for the 14–19 years old, which was substantially lower than for the period until BL, 1.27 [95%-CI 1.90–4.20] for the 80+ and close to unity in other age-groups (Figure 3, indicated by non-solid coloring/gray). For the observation period between FU1 and FU2, we found an SDR overall of 1.14 (95%-CI 1.00–1.32) meaning that most infections in Tirschenreuth were detected and registered by health authorities. Regarding the age-dependent SDR, most age-groups showed a factor around 1 with the elderly population (80+) showing a lower and the young population showing slightly higher factors (e.g., 2.48, 95%-CI 1.59–3.77, for the 14–19 years old) (Figure 3B,E; Supplementary Tables S3 and S4). 

To further investigate the low SDRs in certain strata, especially at FU1, we conducted a sensitivity analysis: in the main analysis we accounted for misclassification in the antibody tests during estimation of the number of new sero-positives. The registered infections may also be affected by misclassification, as they may include individuals who were not infected at the time of testing but were registered as a SARS-CoV-2 case due to a false-positive PCR test result. Such false-positive cases could bias the SDR towards too low values (or even below 1). False-positive PCR tests might play a particularly large role in times of low incidence and large amounts of performed tests (e.g., screening tests among symptom-free persons). To examine the potential magnitude of such a bias, we approximated the total number of performed PCR tests in the county based on the fraction of individuals with self-reported PCR tested in our cohort (BL 12%, BL-FU1 27% and FU1-FU2 46%). We assumed, as a worst-case scenario, a sensitivity of 95% and a specificity of 99.5% for general PCR-testing, derived the fraction of “false-positive infections” among the registered cases (3% until BL, 17% BL-FU1 and 4% FU1-FU2) and corrected the registered cases accordingly. Finally, we calculated SDRs based on our serology data and the corrected registered cases. For BL only a minor increase from 5.35 to 5.51 was observed for overall SDR with slight variations in different age groups. In contrast, the correction had a larger impact at FU 1 increasing SDR from 0.82 to 0.99. Nevertheless, our SDR estimates were still below unity for several age-groups. The change in the FU2 estimate was smaller again, with an overall increase in the SDR from 1.14 to 1.19 (Supplementary Table S7). In conclusion, this sensitivity analysis revealed a potential for a non-negligible impact of “false positive” registered cases during low incidence times. On the other hand, it couldn’t solely explain the finding of SDRs below unity in our cohort, which can most probably be attributed to selection bias in certain age groups.

IFRs for the 3rd observation period were increased compared to IFR in the first or 2nd observation period (i.e., pre-pandemic until BL): while in BL (spring 2020), we found an overall IFR of 2.32% (95%-CI 1.92–2.82), the overall IFR dropped between BL and FU1 to 1.95% (95%-CI 0.96–3.97), but increased between FU1 and FU2 to 3.28% (95%-CI 2.60–4.14), clearly above BL levels (Figure 3C). There was no substantial difference in IFR between men and women. 

We found a distinct age-pattern in IFRs that was similar for all three observation periods: the IFR was increased in the 50–70 years old, highest in the very old (70+), and near 0 in the younger population (<40 years old) (Figure 3C,F; Supplementary Tables S3 and S4). The increased IFR in the third observation period compared to the 1st was due to higher IFR in the 50–70 year old and the very old, not due higher IFR in the young. However, the IFR for higher age-groups in the 3rd observation period was, in fact, the CFR, since the number of expected infected in the county based on serology from this cohort was lower than the number of registered cases.

### 3.6. Contribution of Senior Care Homes to Overall N-Antibody Seropositivity, SDR and IFR across the Observation Periods

**Figure 4:**
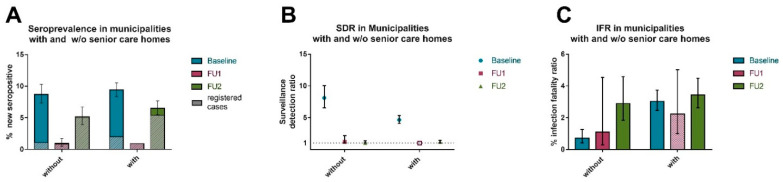
New N-antibody seropositivity, surveillance detection rato (SDR), and infection fatality ratios (IFR) with or without senior care homes (SCH). Shown are (**A**) new seropositive (based on N-antibodies) (%), (**B**) SDR (closed symbols: based on serology data; open symbols: based on registered cases) and (**C**) IFR (%, nonsolid color: based on registered cases) for municipalities with and without senior care homes. Where the expected number of infected in the county based on serology in the cohort was lower than registered cases, the case fatality ratio was used as best approximation of fatality. Error bars represent Wilson 95% confidence intervals (95%-CI) for seroprevalence and 95% Bayesian credibility intervals for SDR and fatality ratio, respectively.

Senior care home residents make up for 1.4% of the county population and form a special group in terms of vulnerability to COVID-19. They contributed for almost 50% of COVID-19 related deaths in individuals 70+ years of age in spring 2020. Despite the offer of a home visit by the study team, participation rate of senior care home residents was low, rendering the calculation of estimates specific to this group unfeasible. As an approximation, we calculated standardized new seropositivity, SDR and IFRs separately for municipalities with and for municipalities without a senior care home. New seropositivity was comparable for all three intervals, BL, BL to FU and FU1 to FU2, respectively. However, at BL, the SDR was 4.66 (95%-CI 4.08–5.32) for municipalities with senior care homes and 8.1 (95%-CI 6.53–10.03) for municipalities without. In contrast, for FU1 and FU2, the SDR dropped down to approximately 1 (min. 1.0 max. 1.19) with no notable differences between municipalities with or without senior care homes. While remarkably higher IFRs were found for municipalities with senior care homes (3.3%; 95-% CI 2.46–3.74) vs municipalities without senior care homes (0.72%; 95%-CI 0.41–1.27) at BL, those differences dwindled in winter 2020/spring 2021 (period between FU1-FU2) with 3.43% (95%-CI 2.63–4.49) for municipalities with and 2.90% (95%-CI 1.84–4.59%) without senior care homes (Figure 4A–C; Supplementary Table S8).

### 3.7. Vaccination

By the end of 2020, different SARS-CoV-2 vaccines received conditional market approval by the EMEA and were shortly after rolled out as part of the national vaccination campaign [1,2,3]. Reflected by 6.06% newly N-antibody seropositves between November 2020 and April 2021 (FU2 compared to FU1), the Tirschenreuth county was once again among the counties in Germany most affected by the quickly spreading SARS-CoV-2 alpha variant. The most affected counties in Germany/Bavaria received extra vaccine doses to accelerate their vaccination campaign.

By combining both N-protein and S-protein based serological tests, together with our questionnaire information on vaccination status, we were able to distinguish infected from vaccinated individuals. Among the 1859 participants in FU2 without report of any received vaccination and successful S- and N-test, 20.19% were N- and S-protein seropositive and thus had experienced a SARS-CoV-2 infection. 1.61% among the non-vaccinated were only S-seropositive, lacking evidence for N-seropositivity (Table 4). This may be attributable to loss of N-specific antibodies, false-negative anti-N-tests, or wrong self-report of vaccination status in the questionnaire. 

Among the 3351 participants at FU2 with valid antibody tests for S and N as well as valid questionnaire information regarding vaccination, 44.3% (*n* = 1492) reported to have been vaccinated at least once. Of these, 63.47% (*n* = 947) were positive for S-specific antibodies and negative for N-specific antibodies. Adding the 137 (9.18%) vaccinated and infected individuals (N and S seropositive) to the 947 (63.47%) vaccinated S-seroconverters, we noticed 27.35% (*n* = 408) vaccinated individuals who scored S antibody negative at the time of the blood draw. We therefore divided the vaccinated into 4 subgroups (once or twice vaccinated, for ≥ or <14 days) assuming that seroconversion is completed 14 days after vaccination. This subgroup analysis revealed 92.12% seroconversion ≥14 days after the 1st vaccination, even rising to 99.67% 14 days after the second vaccination. Most vaccinated but seronegative individuals (*n* = 344) were found among the one times vaccinated, who provided blood <14 days after vaccination (Table 4). 

**Figure 5:**
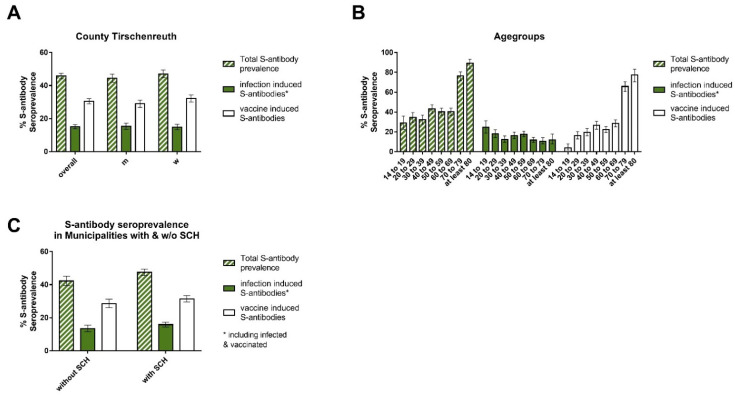
S-specific antibody seroprevalence at FU2. Shown is the standardized S antibody seroprevalence at FU2 (i) counting all S-antibody positive individuals (total prevalence), (ii) counting S-positives that were also N-positive (infection induced antibodies, including vaccinated and unvaccinated individuals) or (iii) counting S-positives that were N-negative (i.e., uninfected but vaccinated individuals), in the overall population of Tirschenreuth (**A**), by age-groups (**B**) and for municipalities with or without senior care homes (**C**).

Although (immune) correlates of protection from SARS-CoV-2 infection or severe disease remain a widely discussed topic, we computed standardized overall S-based seroprevalence within the county of Tirschenreuth. The overall S-based seroprevalence at that time (FU2) was 45.79% (95%-CI 44.11–47.47). Of note, the vaccinated at that time already contributed 30.53% (95%-CI 29.00–32.10). We found no differences between sexes, neither in the vaccinated nor in the naturally infected population, as well as in the combined overall population (Figure 5A, Supplementary Table S9).

Natural S-seropositivity was similar across most age-groups, with a slight trend towards higher S-seropositivity in the younger population. In contrast, vaccine elicted S-antibody seroprevalence revealed a clear increase by higher age, in line with the German vaccine priorisation program assigning a higher priority to the elderly at the start of the roll-out. Thus, vaccine elicted S-seropositivity within the under 20 year old population was very low (3.8%; 95%-CI 1.83–7.73) raising up to 77.0% (95%-CI 70.34–83.11) in the 80+ year olds at the time of FU2, April 2021. Overall S-antibody prevalence reached up to 89.23% (95%-CI 83.54–93.12) in the over 80 years old, when infected (non-vaccinated) individuals were also considered, dropping to about 40% in the 40–69 year old population and dropping further for the younger age groups. Slightly higher natural infection as well as vaccine elicited S-specific seroprevalence was found for municipalities with senior care homes (Figure 5B,C, Supplementary Table S9).

## 4. Discussion

Most longitudinal studies including some nation-wide surveys conducted in several European countries or China [10,11,12,13,34] repeated “cross-sectional analyses” using different panels of individuals, which hampers the comparison over time and may lead to undetected selection bias. In contrast, longitudinal population-based “follow-up analysis” allows for a careful analysis of dropouts and potential selection bias. 

Herein, we conducted a longitudinal population-based study following more than 3300 individuals from the Tirschenreuth county with repeated serology. By this, our serology data captured the SARS-CoV-2 infection occurrence for more than one year since the start of the pandemic and the dynamic over time. Our standardized new seropositivity in each of the three observation periods of 9.2%, 0.9% and 6.1% reflected clearly the high incidence period in the Tirschenreuth county in spring 2020, the low incidence period in summer 2021 and the new surge of infections in autumn/winter 2020/21, respectively, resulting in more than 15% ever seropositives in April 2021. The SDR of about 5 in spring 2020 decreased to unity afterwards, indicating excellent detection of infections owed to massive testing. We still observed a high IFR of > 3% for the 2nd/3rd wave comparable to the 2.3% in the 1st wave. This was again mostly owed to high infection-related mortality in the old aged, but our results indicated a reduced contribution of deaths in senior care homes when compared to the 1st wave. 

Our data document the substantially higher infection occurrence in the Tirschenreuth county compared to other regions in Germany: seroprevalence estimated in November 2020 of at 1.8% in Munich (KoCo19 [16,35]), 1.3–2.8% in 7 different regions in Germany in the MuSPAD study [13], or 1.7% by a nation-wide survey (RKI-SOEP [36]) compared to 9.2% in the Tirschenreuth county. In the MuSPAD study, 4.1% to 13.1% seroprevalence was observed for spring 2021 assessments compared to the 6.5% total seroprevalence estimated in July 2021 in Munich [37,38] and 15.4% ever seropositives in Tirschenreuth after the combined 2nd/3rd wave in April 2021. Despite major differences in the design of these studies, it is fair to conclude that (i) the dynamics of virus spread differs by regions and (ii) the Tirschenreuth population was hit particularly hard both by the 1st and also by the combined 2nd and 3rd wave, in line with national surveillance data in this county (RKI) [39]. 

Of particular relevance is our finding of about 4% of the originally seropositive participants turning seronegative in the re-assessment five months later—both when comparing November 2020 to July 2020 and April 2021 to November 2020. This provides a remarkable consistency of seroconversion from positive to negative regardless of the respective time period under study and a particular strength of our repeated assessments. Although wrong test results (either false positive at preceeding time point or false negative 5 months later) may contribute to the observed changes in sero-status, the loss of N-specific antibodies is the most probable cause [40].

The decrease in SDR from about 5 in the spring 2020 wave to values approaching 1 for the summer/autumn 2020 and winter/spring 2020/21 observation periods underscore the effectiveness of the testing strategy in that county. We observed in several strata of the data from the two observation periods after the baseline survey an even higher number of registered SAR-CoV-2 cases in the county than expected seropositives projected from serology testing in our sample, meaning that the SDR is paradoxically lower than 1 in these strata. In a sensitivity analysis we checked whether this underestimation resulted from false-positive PCR tests which may have inflated the number of registered cases. The results showed that false-positive PCR tests influenced the SDR, especially in the low incidence period during summer/autumn 2020, but had no decisive impact that could explain the underestimation fully. A more plausible explanation is that we must acknowledge some selection bias in our population sample that occurred despite high, though not 100%, participation of randomly drawn subjects. The reasons for the selection bias may be manifold. While we accounted for COVID-19 related deaths often unaccounted for in serology studies, hospitalized infected subjects who are non-participants in the study may contribute to this under-capturing of infected individuals. A more risky behavior towards protecting against infections may be inversely related to participation in a serology—or any health-related—population study. Such differences between participants and non-participants cannot be adjusted for by standardizing for age, sex, and municipality. The high response rate in our study limited, however, the magnitude of the bias. We have not used the raw estimates of seropositives in those strata with SDR < 1, we have instead imputed the number of registered cases for subsequent calculations to further reduce the effect of selection bias on our results. SDRs reported in other German studies like SaarCoPS, MuSPAD and KoCo19 ranged from 2.5 to 4.5 and generally decreased in winter/spring 2020/2021 to 1.3 and 2.9 [13,23,37,39]. However, the previously published studies did not capture a high infection wave in spring 2020 with limited testing available and therefore could not document the change in surveillance detection over time, as achieved in our study here. 

Our observation of an IFR of 3.3% for the 2nd/3rd infection wave versus 2.3% for the 1st wave documents the continued menace of the SARS-Cov-2 infection until a time where the vaccination roll-out was just getting started. The underestimation of the number of SARS-CoV-2 infections in our sample discussed above leads to some overestimation of the IFR. In all instances, where the CFR was lower than the serology-based IFR, we used strata specific CFRs as a better estimate for fatality, which is an upper bound of the fatality estimate. Despite our full efforts to account for bias where possible, some overestimation of the IFR might have remained. 

The overall higher estimate of 3.3% IFR in the winter/spring 2020/21 infection wave compared with the 2.3% estimated for the spring 2020 wave is thus to be interpreted with caution. This is also within statistical uncertainty bounds. Nevertheless, an increased IFR over time—before the vaccination roll-out—would be in line with the introduction of the SARS-CoV-2 alpha variant in December 2020/ January 2021, which was shown to be linked to increased illness and mortality as compared to SARS-CoV2 (wt and D414G) [4,41,42]. 

In our study, we have offered home visits also to senior care home residents and other immobile individuals, to make every possible attempt to capture the full population, also the particularly vulnerable. This might be one reason for IFRs, which seem to be overall higher compared to those reported for other areas in Germany. While SaarCoPS with 2.1% reports an overall IFR close to our baseline result [23], MuSPAD for example reports an IFR of 1.3% across all age groups ranging between 0.3% (lowest; Magdeburg, 11/2020) and 2.4% (highest; Freiburg, 08/2020).The relationship of IFRs during 1st wave and 2nd/3rd wave did not follow a consistent pattern and was higher either for the 1st or the 2nd/3rd wave, dependent on the investigated region, respectively.

Noteworthy was our observation of a reduced impact on fatality from Tirschenreuth county municipalities with senior care homes. These municipalities accounted for a large part of the overall IFR in spring 2020 (IFR of 3.2% with and 0.7% w/o senior care homes), but not anymore in winter/spring 2020/21 (IFR of 3.1% with and 2.6% w/o). This suggests that senior care homes were not prominent drivers of infection fatality during the 2nd and 3rd wave any more. 

Consistent with previous reports [8,43], and also in line with our BL analysis, the proportion of elderly is key for estimated IFRs. IFRs determined for the different age groups at FU2 increased from 0.6% to 3.5%, 10.6% and 24.2% for the 50–59, the 60–69, the 70–79 and the 80+ years old participants, respectively. Trends to higher IFRs at FU2 as compared to the BL analysis were again within statistical uncertainty bounds and may also represent an overestimate for the reasons discussed above. Our age-group specific IFR estimates are in general agreement with several pre-vaccination metaanalysis reporting an exponential relationship between age and IFR for COVID-19 [8,43]. Substantial heterogeneity was found in the IFR by age, location, and time, respectively. Age-specific IFR estimates increased through ages 30 years (0.05%), 60 years (1%) and 90 years (20.3%). Global IFR estimates including all ages are lower at 0.3% in January 2021 [43], as is the proportion of high aged individuals compared to Germany. 

We also found an exceptionally high early response to the vaccination campaign in the elderly in the Tirschenreuth county, leading (together with previous infections) to an S-antibody seroprevalence of 76.3% and 89.2% in the 70–79 years old or >80 years old population, respectively, until April 2021. Unfortunately, no real vaccination tracking was applied in Germany. Nevertheless, estimates of 20% overall (basic) immunization rate in Germany in the beginning of June 2021 [44] support our findings of a higher, but realistic, overall immunization rate of 30.5% for the Tirschenreuth population at that time. 

Among the strengths of our study are the population-based, prospective cohort design and the large number of participants across the various age-groups combined with high retention of study subjects over the complete follow-up period. This design allowed us to systematically describe the evolution of seroprevalence in our population over time. More specifically, we were able to describe the frequency of newly emerging seropositivity and the loss of existing antibodies, rather than just providing information on seroprevalence at specific time points. 

In our study, we observed an association between prior serostatus and willingness to participate in further assessments; seropositive study participants were more likely to participate in the further assessments. A reason for this could be the disclosure of test results to study participants, which in principle could also be waived. However, we think that such disclosure can act as an important incentive for study participation and—at times when no vaccination was available—has been perceived as obligation for ethical reasons. By performing the sequential estimation of seroprevalence at each time point, we can avoid bias due to this differential response. In our case, the (unstandardized) cross-sectional estimate of overall seroprevalence was, e.g., 15.69% in FU2, while the estimate from the sequential approach was 14.09%. Magnitude of the bias of the cross-sectional estimate depends on the degree of differential response due to knowledge of the serostatus at previous time points. In case of a seroprevalence study with prospective cohort design and disclosure of antibody test results at intermediate study time points we recommend to focus on cumulative incidence of (new) seropositivity at each study time point to characterize infection dynamics and use the sequential approach when interested in seroprevalence at specific study time points.

We also acknowledge some limitations, that might reflect general challenges of seroprevalence studies. While seroprevalence studies are superior to registered infection data to estimate cumulative infections by the detectability of asymptomatic infections, selection bias in the beginning as well as differential attrition at follow-up are important to consider when interpreting results. Our drop-out analyses identified lower attrition among individuals having been seropositive at baseline. This might have derived from the fact, that study participants were informed about their serostatus and seropositives might have been more curious to participate further in line with previous reports of enrichment of previously tested positive individuals among NAKO participants [45]. We were able to account for this by the sequential assessment of period-specific incidence rather than comparing directly computed total seropositivity at each assessment. The observation of lower number of serology-based expected than registered infected individuals and its consequences is duly noted above. Overall, seroprevalence studies and derived SDRs and IFR are important to help judge the pandemic dynamic, particularly when applied longitudinally, but our in-depth analyses also highlights some underlying challenges.

## 5. Conclusions

Our population-based, prospective cohort design and the large number of participants across the various age-groups combined with high retention of study subjects over the complete follow-up period allowed us to systematically describe the evolution of seroprevalence, SDR and IFR in our population over time. Along these lines, decreased underreporting of infections highlights the success of massively increased testing in the population. A persistent high IFR particularly among the old aged was documented for the combined 2nd and 3rd infection wave in winter/spring 2020/21 before the vaccination roll-out. Though elderly were still at highest mortality risk, we provide evidence that senior care homes were no longer the dominant key determinants of infection fatality during the 2nd and 3rd wave. High vaccination rates especially in individuals aged 70+ were noticed suggesting good compliance of this risk group in that county with the implemented vaccination campaign. 

## Figures and Tables

**Table 1:** Participant characteristics. Shown are, as indicated, median and IQR or percentage and number of individuals for the given characteristic for analyzable participants at BL, FU1, and FU2.

Variable	BL [Participants]	FU_1 [Participants]	FU_2 [Participants]
**Age** **median (min, max, IQR)**	52.0 (14.0, 102.0, 35.0–64.0) [*n* = 4181]	53.0 (14.0, 102.0, 37.0–64.0) [*n* = 3513]	53.0 (14.0, 102.0, 37.0–64.0) [*n* = 3374]
**Age 14–20** **% (*n*)**	5.4 (225) [*n* = 4181]	5.0 (176) [*n* = 3513]	5.2 (177) [*n* = 3374]
**Age 20–49** **% (*n*)**	40.8 (1707) [*n* = 4181]	38.3 (1345) [*n* = 3513]	38.1 (1284) [*n* = 3374]
**Age 50–69** **% (*n*)**	38.8 (1624) [*n* = 4181]	41.2 (1449) [*n* = 3513]	41.2 (1389) [*n* = 3374]
**Age 70+** **% (*n*)**	14.9 (625) [*n* = 4181]	15.5 (543) [*n* = 3513]	15.5 (524) [*n* = 3374]
**Female** **% (*n*)**	51.6 (2158) [*n* = 4181]	53.0 (1861) [*n* = 3513]	53.7 (1813) [*n* = 3374]
**BMI** **median (min, max, IQR)**	26.6 (13.9, 62.1, 23.7–30.4) [*n* = 4134]	26.6 (13.9, 62.1, 23.7–30.3) [*n* = 3474]	26.6 (13.9, 62.1, 23.7–30.4) [*n* = 3339]
**Disease ^1^**			
**autoimmune** **% (*n*)**	7.1 (289) [*n* = 4081]	7.3 (250) [*n* = 3435]	7.4 (243) [*n* = 3300]
**cancer** **% (*n*)**	4.9 (202) [*n* = 4081]	5.2 (178) [*n* = 3435]	5.0 (164) [*n* = 3300]
**diabetes** **% (*n*)**	7.6 (312) [*n* = 4081]	7.5 (259) [*n* = 3435]	7.4 (245) [*n* = 3300]
**cardiovascular** **% (*n*)**	9.9 (402) [*n* = 4081]	9.6 (331) [*n* = 3435]	9.5 (314) [*n* = 3300]
**none ^4^** **% (*n*)**	75.8 (3093) [*n* = 4081]	75.6 (2596) [*n* = 3435]	76.0 (2507) [*n* = 3300]
**Education**			
**Years ^2^** **median (min, max, IQR)**	11.0 (6.0, 22.0, 10.0–14.0) [*n* = 4085]	11.0 (6.0, 22.0, 10.0–13.0) [*n* = 3433]	11.0 (6.0, 22.0, 10.0–14.0) [*n* = 3301]
**High ^3^** **% (*n*)**	30.0 (1225) [*n* = 4085]	29.5 (1013) [*n* = 3433]	29.8 (985) [*n* = 3301]
**antibody status**			
**N-antibody positive at BL** **% (*n*)**	8.9 (374) [*n* = 4181]	10.0 (351) [*n* = 3513]	10.3 (349) [*n* = 3374]

^1^ diseases = self-reported disease from questionnaire; ^2^ education years = years of schooling and university/vocational training; ^3^ high education ≥12 education years; ^4^ no autoimmune disease, no cancer, no diabetes, no cardiovascular diseases as per self-report.

**Table 2:** Crude N-test seropositivity among analyzed participants and changes over time. Shown are the number of total N-antibody positives, newly seropositive, and newly seronegative participants for N-protein specific antibodies at baseline (June 2020), at FU1 (November 2020), and at FU2 (April 2021). Also shown is the percentage of the according participants group as well as ever N-seropositives and total N-seropositives.

Time of Analysis Sex	AnalyzableParticipants ^1^ *n*	Total N Antibody Positive % (*n*) ^2^	Analyzable Participants *n* Previously Pos/Neg	Newly N Antibody Positive % (*n*) ^3^	Newly N Antibody Negative % (*n*) ^4^	Ever Seropositive (%) ^5^	Total N Antibody Positives (%) ^5^
**Baseline**	4181	8.95 (374)	0/4181	8.95 (374)	(n/a)		
women	2158	9.08 (196)	0/2158	9.08 (196)	(n/a)		
men	2023	8.80 (178)	0/2023	8.8 (178)	(n/a)		
**FU1**	3513	10.22 (359)	351/3162	0.66 (21)	3.70 (13)	9.55	9.22
women	1861	10.26 (191)	187/1674	0.66 (11)	3.74 (7)	9.54	9.21
men	1652	10.17 (168)	164/1488	0.67 (10)	3.66 (6)	9.58	9.23
**FU2**	3177	15.68 (498)	349/2828	5.80 (164)	4.30 (15)	14.80	14.09
women	1710	15.56 (266)	186/1524	5.91 (90)	5.38 (10)	14.89	14.08
men	1467	15.81 (232)	163/1304	5.67 (74)	3.07 (5)	14.69	14.10

^1^ analyzable for the respective observation period and all periods before. ^2^ derived directly from antibodies at BL, FU1, or FU2, respectively ^3^ compared to previously negative. ^4^ compared to previously positive. ^5^ derived from the sequential approach (Methods).

**Table 3:** PCR-test report. Via questionnaire, 66, 19 and 153 participants reported a positive PCR-test for the period until BL, between BL and FU1, or between FU1 and FU2, respectively, which were confirmed by health authorities. We derived the % N-antibody negatives and N-antibody positives at BL, FU1, FU2, respectively, for these individuals. Additionally, we derived the % N-antibody negatives and N-antibody positives at FU2 for all individuals with self-reported positive PCR-test (health authority confirmed) at any time until FU2 assessment (April 2021).

Timepoint	# Confirmed Positive Registered PCR Test (# Total Self-Reported PCR Tests)	N-Antibody Negative # (%[Group]; %[All Tests])	N-Antibody Positive # (%[Group]; %[All Tests])
Until BL 12/2019–4/2020	*n* = 66 (*n* = 501)	4 (6.06; 0.80)	62 (93.94; 12.38)
between BL and FU1 6/2020–11/2020	*n* = 19 (*n* = 1064)	6 (31.58; 0.56)	13 (68.4; 1.22)
between FU1 and FU2 11/2020–4/2021	*n* = 153 (*n* = 1568)	8 (5.23; 0.51)	145 (94.77; 9.25)
until FU2 12/2019–4/2021	*n* = 238 (*n* = 3133)	18 (7.56; 0.57)	220 (92.44; 7.02)

**Table 4:** Antibody response in vaccinated (self reported) and infected study participants. For the 3351 individuals with a valid questionnaire result for vaccination at FU2, we determined S- and N-antibody seropositivity. Indicated are % (#) of S antibody positive and % (#) N antibody positive among unvaccinated and vaccinated at FU2 (April 2021, based on self-report via questionnaire). We determined the impact of 1 versus 2 vaccinations and also the time interval from vaccination to blood drawl (< versus ≥ 14 days after vaccination).

Vaccination ^1^- and Serostatus	N + S	Only S	S	S
	Antibody Positive	Antibody Positive	Seropositive	Antibody Negative
	% (#)	% (#)	% (#)	% (#)
Not vaccinated,	20.17 (375)	1.61 (30)	21.79 (405)	78.21 (1454)
*n* = 1859				
vaccinated (all),	9.18 (137)	63.47 (947)	72.65 (1084)	27.35 (408)
*n* = 1492				
1× vaccinated (<14 d),	8.48 (38)	14.73 (66)	23.21 (104)	76.79 (344)
*n* = 448				
1× vaccinated (>14 d),	10.69 (76)	81.43 (579)	92.12 (655)	7.88 (56)
*n* = 711				
2× vaccinated (<14 d),	25 (2)	75 (6)	100 (8)	0 (0)
*n* = 8				
2× vaccinated (>14 d),	6.21 (19)	93.46 (286)	99.67 (305)	0.33 (1)
*n* = 306				
No of vaccinations unknown	10.5 (2)	52.6 (10)	63.2 (12)	36.8 (7)
*n* = 19				

^1^ according to questionnaire. For *n* = 23 individuals either vaccine or serostatus couldn’t be determined and they were therefore excluded from the analysis.

## Data Availability

All authors declare that data and materials will be made available according to the guidelines of the journal.

## Supplementary Materials

The following are available online at www.mdpi.com/article/10.3390/v14061168/s1: Suppl. Table S1: (Supplement to Table 1) Participant characteristics and dropout analysis. Suppl. Table S2: (Supplement to Table 2). Crude seroprevalence among analyzed participants and changes over time. Suppl. Table S3: (supplement to Figure 3). Standardized (N-based) seroprevalence, surveillance detection ratio (SDR), and infection fatality ratios (IFR) overall and by age groups for Baseline (BL), Follow up 1 (BL to FU1) and Follow up 2 (FU1 to FU2); Suppl. Table S4: (supplement to Figure 3, part 2) Numbers of registered SARS-CoV-2 infections and related death case in the county Tirschenreuth.; Suppl. Table S5: (Supplement to Supplementary Figure S1) Standardized % “ever seropositives”; Suppl. Table S6: Standardized (N-based) period seroprevalence by municipality at Baseline (BL), at FU1 (period between BL and FU1) and FU2 (period between FU1 and FU2).; Suppl. Table S7: Sensitivity analysis of SDRs; Suppl. Table S8: (Supplement to Figure 4) Standardized (N-based) Seroprevalence, SDR and IFR in municipalities with and w/o SCH at Baseline (BL), at FU1 (period between BL and FU1) and FU2 (period between FU1 and FU2).; Suppl. Table S9: (Supplement to Figure 5) Standardized (S-based) period seroprevalence at Baseline (BL), at FU1 (period between BL and FU1) and FU2 (period between FU1 and FU2). Suppl. Figure S1: Cumulative standardized (N-based) Seroprevalence overall and by age groups for Baseline (BL), Follow up 1 (FU1) and Follow up 2 (FU2).
