# Supplementary material for: Time Trend in SARS-CoV-2 Seropositivity, Surveillance Detection- and Infection Fatality Ratio until Spring 2021 in the Tirschenreuth County—Results from a Population-Based Longitudinal Study in Germany
Source: Viruses. 2022 May 27;14(6):1168. doi: 10.3390/v14061168 (PMC9228731; doi:10.3390/v14061168)
Supplement: Supplementary file 1 [file viruses-14-01168-s001.zip › viruses-1680607-Supplementary Figure S1 Standardized ever seropositives.pdf]

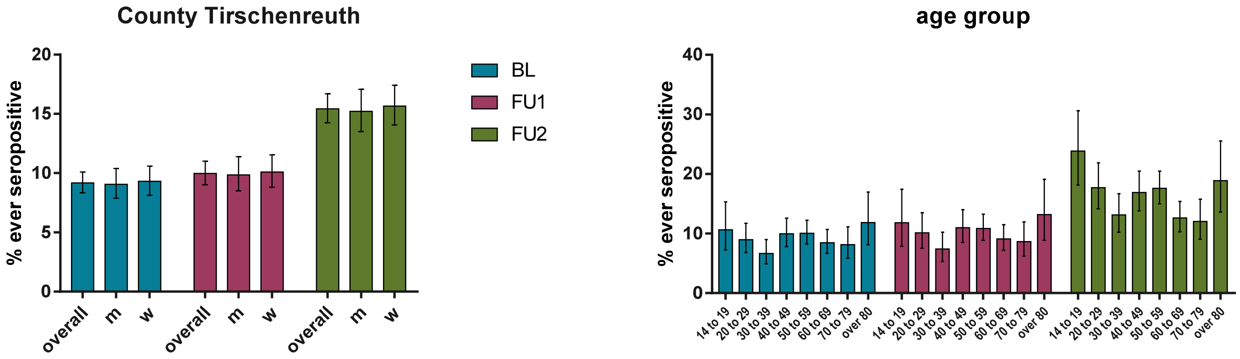

Supplementary Figure S1 Standardized ever seropositives (N-based) overall and by age groups until Baseline (BL), Follow up 1 (FU1) and Follow up 2 (FU2)
