# Supplementary material for: Time Trend in SARS-CoV-2 Seropositivity, Surveillance Detection- and Infection Fatality Ratio until Spring 2021 in the Tirschenreuth County—Results from a Population-Based Longitudinal Study in Germany
Source: Viruses. 2022 May 27;14(6):1168. doi: 10.3390/v14061168 (PMC9228731; doi:10.3390/v14061168)
Supplement: Supplementary file 1 [file viruses-14-01168-s001.zip › viruses-1680607-Supplementary-Tables.pdf]

Supplemental Table S1 (Supplement to Table 1) Participant characteristics and dropout analysis. *Shown are, as indicated, median and IQR or percentage and number of individuals for the given characteristic for analyzable participants and drop-outs at BL, FU1, and FU2. P-values test for difference in the characteristics between analyzable participants and drop-outs.*

Variable	BL	FU1 participant	FU1 dropout	FU1 p-value	FU2 participant	FU2 dropout	FU2 p-value
**Age** median (min, max, IQR)	52.0 (14.0, 102.0, 35.0-64.0) [n=4181]	53.0 (14.0, 102.0, 37.0-64.0) [n=3513]	42.0 (14.0, 93.0, 29.0-58.0) [n=668]	<0.001	53.0 (14.0, 102.0, 37.0-64.0) [n=3374]	44.0 (14.0, 91.0, 30.0-59.0) [n=807]	<0.001
**Age 14-20** % (n)	5.4 (225) [n=4181]	5.0 (176) [n=3513]	7.3 (49) [n=668]	0.019	5.2 (177) [n=3374]	5.9 (48) [n=807]	0.48
**Age 20-49** % (n)	40.8 (1707) [n=4181]	38.3 (1345) [n=3513]	54.2 (362) [n=668]	<0.001	38.1 (1284) [n=3374]	52.4 (423) [n=807]	<0.001
**Age 50-69** % (n)	38.8 (1624) [n=4181]	41.2 (1449) [n=3513]	26.2 (175) [n=668]	<0.001	41.2 (1389) [n=3374]	29.1 (235) [n=807]	<0.001
**Age 70+** % (n)	14.9 (625) [n=4181]	15.5 (543) [n=3513]	12.3 (82) [n=668]	0.04	15.5 (524) [n=3374]	12.5 (101) [n=807]	0.035
**Female** % (n)	51.6 (2158) [n=4181]	53.0 (1861) [n=3513]	44.5 (297) [n=668]	<0.001	53.7 (1813) [n=3374]	42.8 (345) [n=807]	<0.001
**BMI** median (min, max, IQR)	26.6 (13.9, 62.1, 23.7-30.4) [n=4134]	26.6 (13.9, 62.1, 23.7-30.3) [n=3474]	26.8 (16.0, 56.5, 23.9-30.4) [n=660]	0.396	26.6 (13.9, 62.1, 23.7-30.4) [n=3339]	26.3 (16.0, 56.5, 23.7-30.1) [n=795]	0.556
**Disease**							
**autoimmune** % (n)	7.1 (289) [n=4081]	7.3 (250) [n=3435]	6.0 (39) [n=646]	0.296	7.4 (243) [n=3300]	5.9 (46) [n=781]	0.172
**cancer** % (n)	4.9 (202) [n=4081]	5.2 (178) [n=3435]	3.7 (24) [n=646]	0.139	5.0 (164) [n=3300]	4.9 (38) [n=781]	0.977
**diabetes** % (n)	7.6 (312) [n=4081]	7.5 (259) [n=3435]	8.2 (53) [n=646]	0.615	7.4 (245) [n=3300]	8.6 (67) [n=781]	0.309
**cardiovascular** % (n)	9.9 (402) [n=4081]	9.6 (331) [n=3435]	11.0 (71) [n=646]	0.323	9.5 (314) [n=3300]	11.3 (88) [n=781]	0.158
**none** % (n)	75.8 (3093) [n=4081]	75.6 (2596) [n=3435]	76.9 (497) [n=646]	0.49	76.0 (2507) [n=3300]	75.0 (586) [n=781]	0.614
**Education**							
**years** median (min, max, IQR)	11.0 (6.0, 22.0, 10.0-14.0) [n=4085]	11.0 (6.0, 22.0, 10.0-13.0) [n=3433]	11.0 (6.0, 21.0, 10.0-14.0) [n=652]	0.406	11.0 (6.0, 22.0, 10.0-14.0) [n=3301]	11.0 (6.0, 21.0, 10.0-14.0) [n=784]	0.365
**high** % (n)	30.0 (1225) [n=4085]	29.5 (1013) [n=3433]	32.5 (212) [n=652]	0.136	29.8 (985) [n=3301]	30.6 (240) [n=784]	0.703
**antibody status**							
**N-antibody positive at BL** % (n)	8.9 (374) [n=4181]	10.0 (351) [n=3513]	3.4 (23) [n=668]	<0.001	10.3 (349) [n=3374]	3.1 (25) [n=807]	<0.001

Supplemental Table S2 (Supplement to Table 2). *Crude seroprevalence among analyzed participants and changes over time.* *The number of total and newly seropositive and newly seronegative participants for N or S protein specific antibodies at baseline (June 2020), at FU1 (November 2020), and at FU2 (April 2021) also shown is the percentage of the according participants group.*

Time of analysis<br>participant age	Analyzable participants #	Participants# previously pos/neg	Newly N antibody positive % (#)1	Newly N antibody negative % (#)1	Seropositive as sum	Ever seropositive
**Baseline**	**4181**	**0/4181**	**8.95 (374)**	**(n/a)**		
14 to 19	225	0/225	10.67 (24)	(n/a)		
20 to 29	523	0/523	8.99 (47)			
30 to 39	583	0/583	6.69 (39)			
40 to 49	601	0/601	9.98 (60)			
50 to 59	875	0/875	10.06 (88)			
60 to 69	749	0/749	8.41 (63)			
70 to 79	420	0/420	7.62 (32)			
over 80	205	0/205	10.24 (21)			
**FU1[1]**	**3513**	**351/3162**	**0.66 (21)**	**3.70 (13)**	**9.22**	**9.55**
14 to 19	176	24/152	1.32 (2)	4.17 (1)	11.40	11.84
20 to 29	400	42/358	0.84 (3)	2.38 (1)	9.54	9.75
30 to 39	447	37/410	0.00 (0)	8.11 (3)	6.15	6.69
40 to 49	498	56/442	1.13 (5)	5.36 (3)	10.47	11
50 to 59	766	81/685	0.73 (5)	1.23 (1)	10.59	10.71
60 to 69	683	61/622	0.32 (2)	3.28 (2)	8.43	8.71
70 to 79	373	32/341	0.59 (2)	6.25 (2)	7.68	8.16
over 80	170	18/152	1.32 (2)	0.00 (0)	11.42	11.42
**FU2[2]**	**3177**	**349/2828**	**5.80 (164)**	**4.30 (15)**	**14.09**	**14.80**
14 to 19	164	24/140	13.57 (19)	4.17 (1)	22.95	23.81
20 to 29	340	41/299	8.36 (25)	4.88 (2)	16.63	17.30
30 to 39	390	32/358	6.15 (22)	12.5 (4)	11.15	12.42
40 to 49	454	57/397	5.79 (23)	8.77 (5)	14.74	16.16
50 to 59	700	83/617	7.46 (46)	2.41 (2)	17.00	17.37
60 to 69	629	60/569	3.51 (20)	1.67 (1)	11.51	11.91
70 to 79	348	32/316	2.53 (8)	0.00 (0)	10.02	10.49
over 80	142	20/132	0.76 (1)	0.00 (0)	12.1	12.1

Suppl Table S3 (supplement to figure 2). Standardized (N-based) seroprevalence, surveillance detection ratio (SDR), and infection fatality ratios (IFR) overall and by age groups for Baseline (BL), Follow up 1 (BL to FU1) and Follow up 2 (FU1 to FU2). Shown are the population at risk in the general Tirschenreuth population and the analyzed cohort as numbers and percentage of the overall group. Further shown are standardized and corrected seroprevalence (based on N-antibodies) (%), the surveillance detection ratio (SDR) and infection fatality ratio (%) in the overall county population, the indicated sexes and the indicated age groups. Further given are the 95% Wilson confidence intervals (95%-CI) or 95% Bayesian credibility intervals, respectively. The CI is marked as [reg.] for seroprevalence and SDR, when case prevalence based on registered infections exceeded standardized seroprevalence. In those cases SDR was set to 1.0 and registered case numbers were used for further calculations.

Sub group	Population at risk Tirschenreuth #; [%]			Population at risk Cohort #; [%]			standardized new seropositive %; [95%-CI]			SDR ratio; [95% CI]			IFR %; [95%-CI]		
	**BL**	**FU1**	**FU2**	**BL**	**FU1**	**FU2**	**BL**	**FU1**	**FU2**	**BL**	**FU1**	**FU2**	**BL**	**FU1**	**FU2**
**Overall**	64643; [100]	58709; [100]	58288; [100]	4181; [100]	3162; [100]	2828; [100]	9.18; [8.34 - 10.09]	0.87; [reg.]	6.06; [5.24 - 7.00]	5.35; [4.78 - 5.99]	1.00; [reg.]	1.14; [1.00 - 1.32]	2.32; [1.92 - 2.82]	1.95; [0.96 - 3.97]	3.28; [2.60 - 4.14]
**m**	32239; [49.87]	29318; [49.94]	29105; [49.93]	2023; [48.39]	1488; [47.06]	1304; [46.11]	9.06; [7.89 - 10.39]	0.88; [reg.]	5.94; [4.78 - 7.35]	6.29; [5.32 - 7.41]	1.00; [reg.]	1.17; [1.00 - 1.46]	2.64; [2.04 - 3.43]	1.95; [0.72 - 5.31]	3.18; [2.27 - 4.47]
**w**	32404; [50.13]	29392; [50.06]	29183; [50.07]	2158; [51.61]	1674; [52.94]	1524; [53.89]	9.30; [8.14 - 10.59]	0.87; [reg.]	6.19; [5.09 - 7.51]	4.68; [4.02 - 5.44]	1.00; [reg.]	1.11; [1.00 - 1.36]	2.02; [1.53 - 2.68]	1.95; [0.74 - 5.26]	3.38; [2.46 - 4.65]
**age group**															
**14 to 19**	3994; [6.18]	3570; [6.08]	3522; [6.04]	225; [5.38]	152; [4.81]	140; [4.95]	10.62; [7.24 - 15.33]	1.33; [0.37 - 4.69]	13.64; [8.92 - 20.30]	11.51; [6.97 - 18.75]	1.44; [1.00 - 5.31]	2.48; [1.59 - 3.77]	0.00; [0.00 - 0.90]	0.00; [0.00 - 10.36]	0.00; [0.00 - 0.80]
**20 to 29**	8146; [12.60]	7416; [12.63]	7354; [12.62]	523; [12.51]	358; [11.32]	299; [10.57]	8.96; [6.80 - 11.72]	1.28; [reg.]	8.39; [5.76 - 12.09]	7.39; [5.27 - 10.30]	1.00; [reg.]	1.44; [1.00 - 2.10]	0.14; [0.03 - 0.78]	0.00; [0.00 - 4.49]	0.00; [0.00 - 0.62]
**30 to 39**	8430; [13.04]	7869; [13.40]	7869; [13.50]	583; [13.94]	410; [12.97]	358; [12.66]	6.65; [4.90 - 8.98]	0.80; [reg.]	6.16; [4.11 - 9.15]	5.58; [3.90 - 7.97]	1.00; [reg.]	1.14; [1.00 - 1.70]	0.00; [0.00 - 0.67]	0.00; [0.00 - 7.17]	0.00; [0.00 - 0.79]
**40 to 49**	8782; [13.59]	7909; [13.47]	7819; [13.41]	601; [14.37]	442; [13.98]	397; [14.04]	9.94; [7.80 - 12.59]	1.14; [0.49 - 2.63]	6.65; [reg.]	5.62; [4.21 - 7.45]	1.29; [1.00 - 3.06]	1.00; [reg.]	0.00; [0.00 - 0.43]	0.00; [0.00 - 4.71]	0.19; [0.04 - 1.12]
**50 to 59**	12813; [19.82]	11524; [19.63]	11440; [19.63]	875; [20.93]	685; [21.66]	617; [21.82]	10.06; [8.24 - 12.23]	0.89; [reg.]	7.53; [5.70 - 9.88]	6.02; [4.74 - 7.61]	1.00; [reg.]	1.36; [1.02 - 1.80]	0.47; [0.21 - 1.04]	0.00; [0.00 - 4.08]	0.58; [0.25 - 1.42]
**60 to 69**	10412; [16.11]	9531; [16.23]	9501; [16.3]	749; [17.91]	622; [19.67]	569; [20.12]	8.46; [6.67 - 10.67]	0.69; [reg.]	3.86; [reg.]	6.30; [4.73 - 8.39]	1.00; [reg.]	1.00; [reg.]	1.03; [0.53 - 2.04]	1.52; [0.28 - 10.61]	3.54; [1.81 - 6.95]
**70 to 79**	6772; [10.48]	6224; [10.60]	6186; [10.61]	420; [10.05]	341; [10.78]	316; [11.17]	8.10; [5.85 - 11.10]	0.60; [0.17 - 2.13]	3.67; [reg.]	3.78; [2.62 - 5.39]	1.28; [1.00 - 4.79]	1.00; [reg.]	6.38; [4.04 - 10.16]	2.69; [0.40 - 22.63]	10.57; [5.37 - 20.96]
**at least 80**	5294; [8.19]	4667; [7.95]	4597; [7.89]	205; [4.90]	152; [4.81]	132; [4.67]	11.84; [8.11 - 16.99]	1.50; [0.44 - 4.95]	6.57; [reg.]	2.84; [1.90 - 4.20]	1.27; [1.00 - 4.29]	1.00; [reg.]	14.04; [9.23 - 21.63]	11.43; [2.90 - 42.39]	24.17; [12.55 - 47.61]

Suppl Table S4 (supplement to figure 2, part 2).

Numbers of registered SARS-CoV-2 infections and related death case in the county Tirschenreuths. *Given are the general population of Tirschenreuth as of the census 2019 (14 years and older) as well as registered deaths andSARS-CoV-2 infections for BL, the period beween BL and FU1 (FU1) and the period between FU1 and FU2 (FU2). Numbers are shown for the overall county population as well as the indicated age groups, sexes and municipalities.*

	**Tirschenreuth population #**	**Registered deaths #**			**Registered infections #**		
		**BL**	**FU1**	**FU2**	**BL**	**FU1**	**FU2**
**overall**	64643	138	10	116	1109	513	3100
**age group**							
14 to 19	3994	0	0	0	37	33	194
20 to 29	8146	1	0	0	99	95	428
30 to 39	8430	0	0	0	101	63	427
40 to 49	8782	0	0	1	156	70	520
50 to 59	12813	6	0	5	214	102	635
60 to 69	10412	9	1	13	139	66	367
70 to 79	6772	35	1	24	145	29	227
over 80	5294	87	8	73	218	55	302
**sex**							
m	32239	77	5	55	464	257	1473
w	32404	61	5	61	645	256	1627
**municipality**							
Bad Neualbenreuth	1186	0	0	5	10	4	55
Brand	1025	0	0	1	5	2	65
Bärnau	2795	2	0	4	45	33	117
Ebnath	1710	0	0	0	3	7	52
Erbendorf	4476	21	0	6	93	22	187
Falkenberg	822	0	0	2	11	3	38
Friedenfels	1103	1	1	1	19	2	53
Fuchsmühl	1387	8	0	0	54	5	59
Immenreuth	1600	0	0	2	1	17	132
Kastl	1208	1	0	0	5	10	50
Kemnath	4773	7	0	4	30	11	307
Konnersreuth	1501	9	0	3	60	14	58
Krummennaab	1299	2	0	0	34	8	36
Kulmain	1928	0	0	2	4	19	93
Leonberg	870	2	0	2	12	6	37
Mitterteich	5899	21	0	17	172	60	191
Mähring	1560	4	0	4	31	17	64
Neusorg	1810	2	1	4	17	16	77
Pechbrunn	1173	0	0	1	20	8	30
Plößberg	2835	15	0	1	85	21	151
Pullenreuth	1500	1	0	2	7	7	78
Reuth b.Erbendorf	994	0	1	2	16	12	50
Tirschenreuth	7807	15	5	30	145	80	516
Waldershof	3869	12	2	5	51	42	176
Waldsassen	5862	8	0	9	103	65	293
Wiesau	3651	7	0	9	75	22	135
**Senior care homes**							
without senior care home	20773	13	2	28	223	155	950
with senior care home	43870	125	8	88	885	358	2150

Supplemental Table S5 (Supplement to Supplemental Figure 2)

Standardized % "ever seropositives" for the indicated intervals and subgroups

	**standardized ever seropositive %; [CI]**		
	**until BL**	**until FU1**	**until FU2**
**Overall**	9.18; [8.34-10.09]	9.97; [9.03-11.01]	15.43; [14.25-16.69]
**m**	9.06; [7.89-10.39]	9.86; [8.51-11.39]	15.21; [13.51-17.08]
**w**	9.30; [8.14-10.59]	10.09; [8.80-11.54]	15.65; [14.05-17.40]
**Age group**			
14 to 19	10.62; [7.24-15.33]	11.81; [7.84-17.41]	23.84; [18.16-30.63]
20 to 29	8.96; [6.80-11.72]	10.13; [7.54-13.47]	17.67; [14.14-21.86]
30 to 39	6.65; [4.90-8.98]	7.40; [5.32-10.21]	13.11; [10.22-16.67]
40 to 49	9.94; [7.80-12.59]	10.97; [8.52-14.02]	16.89; [13.83-20.47]
50 to 59	10.06; [8.24-12.23]	10.86; [8.85-13.26]	17.57; [14.99-20.48]
60 to 69	8.46; [6.67-10.67]	9.09; [7.16-11.48]	12.60; [10.27-15.37]
70 to 79	8.10; [5.85-11.10]	8.65; [6.20-11.94]	12.00; [9.04-15.76]
at least 80	11.84; [8.11-16.99]	13.17; [8.89-19.07]	18.87; [13.62-25.55]
**Municipality**			
Bad Neualbenreuth	7.65; [3.77-14.88]	7.98; [3.73-16.29]	14.07; [8.09-23.34]
Brand	5.16; [1.78-14.02]	5.35; [1.73-15.34]	14.00; [6.95-26.19]
Bärnau	9.51; [6.06-14.63]	12.25; [8.02-18.28]	16.44; [11.35-23.22]
Ebnath	6.25; [2.47-14.91]	8.20; [3.38-18.58]	11.24; [5.24-22.48]
Erbendorf	8.22; [5.47-12.19]	8.72; [5.63-13.26]	13.08; [9.12-18.41]
Falkenberg	2.73; [0.76-9.37]	4.40; [1.46-12.50]	11.71; [5.83-22.12]
Friedenfels	5.99; [2.62-13.13]	6.17; [2.52-14.32]	10.97; [5.56-20.50]
Fuchsmühl	14.61; [8.69-23.52]	14.97; [8.52-24.96]	19.85; [12.01-31.01]
Immenreuth	1.00; [0.18-5.45]	2.06; [0.52-7.85]	10.22; [5.27-18.91]
Kastl	2.52; [0.71-8.52]	3.34; [1.02-10.39]	8.17; [3.69-17.12]
Kemnath	1.88; [0.83-4.17]	2.11; [0.93-4.71]	10.90; [7.49-15.60]
Konnersreuth	12.73; [7.72-20.28]	13.66; [8.28-21.70]	18.24; [11.71-27.29]
Krummennaab	12.26; [6.86-20.96]	13.76; [7.60-23.64]	16.77; [9.69-27.46]
Kulmain	5.22; [2.57-10.34]	6.21; [2.94-12.62]	10.98; [6.24-18.61]
Leonberg	20.88; [12.95-31.87]	21.57; [12.99-33.62]	34.07; [22.96-47.27]
Mitterteich	19.03; [15.38-23.30]	20.05; [16.01-24.80]	25.07; [20.53-30.23]
Mähring	18.32; [12.14-26.71]	19.41; [12.74-28.44]	23.46; [15.97-33.07]
Neusorg	7.09; [3.81-12.81]	8.16; [4.29-14.99]	12.42; [7.28-20.39]
Pechbrunn	12.71; [6.87-22.33]	15.98; [9.06-26.65]	21.89; [12.94-34.57]
Plößberg	7.06; [4.27-11.46]	7.80; [4.57-13.02]	13.13; [8.94-18.87]
Pullenreuth	3.78; [1.50-9.21]	4.25; [1.58-10.92]	9.43; [4.74-17.87]
Reuth b.Erbendorf	22.62; [14.49-33.52]	23.82; [15.06-35.55]	32.83; [22.29-45.42]
Tirschenreuth	10.64; [8.32-13.51]	11.86; [9.22-15.12]	18.47; [15.18-22.27]
Waldershof	3.80; [1.92-7.40]	4.89; [2.54-9.21]	11.63; [7.57-17.46]
Waldsassen	9.04; [6.47-12.48]	10.14; [7.21-14.09]	15.91; [12.11-20.61]
Wiesau	8.62; [5.64-12.97]	9.79; [6.28-14.94]	16.14; [11.51-22.18]
**Senior care homes**			
without senior care home	8.71; [7.34-10.31]	9.59; [8.04-11.41]	14.25; [12.32-16.42]
with senior care home	9.40; [8.37-10.54]	10.22; [9.06-11.51]	16.05; [14.60-17.62]

Supplemental Table S6.

Standardized (N-based) period seroprevalence by municipality at Baseline (BL), at FU1 (period between BL and FU1) and FU2 (period between FU1 and FU2). Shown are the population at risk in the general Tirschenreuth population and the analyzed cohort as absolute numbers and percentages of the according group. Further shown is standardized and corrected seroprevalence (based on N-antibodies) (%) in the indicated municipalities with the 95% Wilson confidence intervals (95%-CI). The CI is marked as [registered] for seroprevalence, when case prevalence exceeded seoprevalence, as SDR was then set to 1.0 and registered cases were used for all calculations.

**Subgroup**	**Population at risk Tirschenreuth #; [%]**			**Population at risk Cohort #; [%]**			**new seropositive %; [95%-CI]**		
**Municipality**	**BL**	**FU1**	**FU2**	**BL**	**FU1**	**FU2**	**BL**	**FU1**	**FU2**
**Bad Neualbenreuth**	1186; [1.83]	1095; [1.87]	1095; [1.88]	93; [2.22]	69; [2.18]	66; [2.33]	7.65; [3.77 - 14.88]	0.37; [registered]	6.61; [2.70 - 15.30]
**Brand**	1025; [1.59]	972; [1.66]	972; [1.67]	59; [1.41]	47; [1.49]	45; [1.59]	5.16; [1.78 - 14.02]	0.21; [registered]	9.14; [3.66 - 21.05]
**Bärnau**	2795; [4.32]	2529; [4.31]	2453; [4.21]	183; [4.38]	141; [4.46]	123; [4.35]	9.51; [6.06 - 14.63]	3.03; [1.22 - 7.33]	4.77; [registered]
**Ebnath**	1710; [2.65]	1603; [2.73]	1570; [2.69]	65; [1.55]	51; [1.61]	49; [1.73]	6.25; [2.47 - 14.91]	2.09; [0.39 - 10.50]	3.31; [registered]
**Erbendorf**	4476; [6.92]	4108; [7.00]	4108; [7.05]	262; [6.27]	195; [6.17]	175; [6.19]	8.22; [5.47 - 12.19]	0.54; [registered]	4.78; [2.48 - 9.03]
**Falkenberg**	822; [1.27]	800; [1.36]	786; [1.35]	74; [1.77]	62; [1.96]	55; [1.94]	2.73; [0.76 - 9.37]	1.71; [0.32 - 8.74]	7.64; [3.08 - 17.74]
**Friedenfels**	1103; [1.71]	1037; [1.77]	1036; [1.78]	86; [2.06]	67; [2.12]	63; [2.23]	5.99; [2.62 - 13.13]	0.19; [registered]	5.12; [registered]
**Fuchsmühl**	1387; [2.15]	1184; [2.02]	1184; [2.03]	87; [2.08]	62; [1.96]	53; [1.87]	14.61; [8.69 - 23.52]	0.42; [registered]	5.75; [1.99 - 15.49]
**Immenreuth**	1600; [2.48]	1584; [2.70]	1584; [2.72]	100; [2.39]	83; [2.62]	75; [2.65]	1.00; [0.18 - 5.45]	1.07; [registered]	8.33; [registered]
**Kastl**	1208; [1.87]	1178; [2.01]	1178; [2.02]	83; [1.99]	70; [2.21]	61; [2.16]	2.52; [0.71 - 8.52]	0.85; [registered]	4.99; [1.72 - 13.59]
**Kemnath**	4773; [7.38]	4683; [7.98]	4683; [8.03]	291; [6.96]	251; [7.94]	217; [7.67]	1.88; [0.83 - 4.17]	0.23; [registered]	8.99; [5.86 - 13.54]
**Konnersreuth**	1501; [2.32]	1310; [2.23]	1310; [2.25]	109; [2.61]	88; [2.78]	80; [2.83]	12.73; [7.72 - 20.28]	1.07; [registered]	5.30; [2.14 - 12.57]
**Krummennaab**	1299; [2.01]	1140; [1.94]	1120; [1.92]	84; [2.01]	62; [1.96]	58; [2.05]	12.26; [6.86 - 20.96]	1.71; [0.32 - 8.74]	3.49; [0.97 - 11.79]
**Kulmain**	1928; [2.98]	1827; [3.11]	1827; [3.13]	136; [3.25]	96; [3.04]	82; [2.9]	5.22; [2.57 - 10.34]	1.04; [registered]	5.09; [registered]
**Leonberg**	870; [1.35]	688; [1.17]	688; [1.18]	69; [1.65]	46; [1.45]	39; [1.38]	20.88; [12.95 - 31.87]	0.87; [registered]	15.95; [7.62 - 30.38]
**Mitterteich**	5899; [9.13]	4777; [8.14]	4716; [8.09]	376; [8.99]	252; [7.97]	223; [7.89]	19.03; [15.38 - 23.30]	1.26; [0.44 - 3.54]	6.28; [3.78 - 10.26]
**Mähring**	1560; [2.41]	1274; [2.17]	1274; [2.19]	107; [2.56]	78; [2.47]	72; [2.55]	18.32; [12.14 - 26.71]	1.33; [registered]	5.02; [registered]
**Neusorg**	1810; [2.8]	1682; [2.86]	1662; [2.85]	131; [3.13]	96; [3.04]	81; [2.86]	7.09; [3.81 - 12.81]	1.16; [0.22 - 5.85]	4.63; [registered]
**Pechbrunn**	1173; [1.81]	1024; [1.74]	986; [1.69]	72; [1.72]	57; [1.8]	44; [1.56]	12.71; [6.87 - 22.33]	3.75; [1.07 - 12.26]	7.03; [2.46 - 18.50]
**Plößberg**	2835; [4.39]	2635; [4.49]	2616; [4.49]	202; [4.83]	149; [4.71]	138; [4.88]	7.06; [4.27 - 11.46]	0.80; [registered]	5.77; [registered]
**Pullenreuth**	1500; [2.32]	1443; [2.46]	1443; [2.48]	109; [2.61]	80; [2.53]	71; [2.51]	3.78; [1.50 - 9.21]	0.49; [registered]	5.40; [registered]
**Reuth b.Erbendorf**	994; [1.54]	769; [1.31]	768; [1.32]	72; [1.72]	49; [1.55]	44; [1.56]	22.62; [14.49 - 33.52]	1.56; [registered]	11.82; [5.24 - 24.53]
**Tirschenreuth**	7807; [12.08]	6976; [11.88]	6881; [11.81]	545; [13.04]	409; [12.93]	374; [13.22]	10.64; [8.32 - 13.51]	1.36; [0.61 - 3.02]	7.50; [registered]

**Waldershof**	3869; [5.99]	3722; [6.34]	3720; [6.38]	203; [4.86]	165; [5.22]	146; [5.16]	3.80; [1.92 - 7.40]	1.13; [registered]	7.09; [3.94 - 12.45]
**Waldsassen**	5862; [9.07]	5332; [9.08]	5332; [9.15]	354; [8.47]	271; [8.57]	244; [8.63]	9.04; [6.47 - 12.48]	1.22; [registered]	6.41; [3.96 - 10.21]
**Wiesau**	3651; [5.65]	3336; [5.68]	3294; [5.65]	229; [5.48]	166; [5.25]	150; [5.30]	8.62; [5.64 - 12.97]	1.27; [0.36 - 4.39]	7.05; [3.93 - 12.30]

Supplemental Table S7 Sensitivity analysis of our SDR estimates for Baseline (BL) Follow up 1 (FU1) and Follow up 2 (FU2), calculated with an assumed (lower bound) specificity of 99.5% for PCR testing and thus registered cases at health authorities. Missing a better estimate, the total number of tests in the county was derived from the fraction tested within our cohort and standardized to the whole population.

	**SURVEILLANCE DETECTION RATIO-SENSITIVITY ANALYSIS**		
**SUBGROUP**	**BL**	**FU1**	**FU2**
	sens,<br>[prev; % change]	sens,<br>[prev; % change]	sens,<br>[prev; % change]
**OVERALL**	5.51,<br>[5.35, 3.07]	0.99,<br>[0.82, 20.42]	1.19,<br>[1.14, 4.17]
**M**	6.49,<br>[6.3, 3.11]	1.00,<br>[0.83, 20.66]	1.22,<br>[1.17, 4.17]
**W**	4.81,<br>[4.67, 3.04]	0.98,<br>[0.81, 20.75]	1.16,<br>[1.11, 4.16]
**14 TO 19**	11.79,<br>[11.47, 2.78]	1.76,<br>[1.44, 22.22]	2.58,<br>[2.48, 4.3]
**20 TO 29**	7.6,<br>[7.37, 3.13]	0.79,<br>[0.65, 20.25]	1.5,<br>[1.44, 4.14]
**30 TO 39**	5.72,<br>[5.55, 3.06]	N/A,<br>[N/A, 0]	1.18,<br>[1.14, 4.15]
**40 TO 49**	5.78,<br>[5.6, 3.31]	1.55,<br>[1.29, 20.69]	0.91,<br>[0.88, 4.21]
**50 TO 59**	6.2,<br>[6.02, 2.88]	0.99,<br>[0.82, 20]	1.41,<br>[1.36, 4.1]
**60 TO 69**	6.52,<br>[6.34, 2.96]	0.55,<br>[0.46, 20]	0.99,<br>[0.95, 4.26]
**70 TO 79**	3.89,<br>[3.78, 2.84]	1.55,<br>[1.28, 20.83]	0.83,<br>[0.79, 4.13]
**AT LEAST 80**	2.97,<br>[2.88, 3.32]	1.56,<br>[1.27, 22.22]	0.37,<br>[0.35, 4.14]

Supplemental Table S8 (Supplement to figure 3).

Standardized (N-based) Seroprevalence, SDR and IFR in municipalities with and w/o SCH at Baseline (BL), at FU1 (period between BL and FU1) and FU2 (period between FU1 and FU2). Shown are the population at risk in the general Tirschenreuth population and the analyzed cohort as absolute numbers and percentages of the according group. Further shown is standardized and corrected seroprevalence (based on N-antibodies) (%) in the indicated municipalities with the 95% Wilson confidence intervals (95%-CI) as well as SDR and IFR with 95% Bayesian credibility intervals. The CI is marked as [reg.] for seroprevalence and SDR, when case prevalence exceeded seoprevalence, as SDR was then set to 1.0 and registered cases were used for all calculations.

Subgroup	Population at risk Tirschenreuth #; [%]			Population at risk Cohort #; [%]			new seropositive %; [95%-CI]			SDR #; [95%-CI]			IFR %; [95%-CI]		
	BL	FU1	FU2	BL	FU1	FU2	BL	FU1	FU2	BL	FU1	FU2	BL	FU1	FU2
without senior care home	20773; [32.13]	18964; [32.30]	18780; [32.22]	1392; [33.29]	1058; [33.46]	947; [33.49]	8.71; [7.34 – 10.31]	0.97; [0.53 – 1.76]	5.15; [3.91 – 6.74]	8.10; [6.53 – 10.03]	1.18; [1.00 – 2.19]	1.02; [1.00 – 1.34]	0.72; [0.41 – 1.27]	1.09; [0.29 – 4.53]	2.90; [1.84 – 4.59]
with senior care home	43870; [67.87]	39746; [67.70]	39508; [67.78]	2789; [66.71]	2104; [66.54]	1881; [66.51]	9.40; [8.37 – 10.54]	0.90; [reg.]	6.50; [5.47 – 7.70]	4.66; [4.08 – 5.32]	1.00; [reg.]	1.19; [1.00 – 1.42]	3.03; [2.46 – 3.74]	2.23; [1.00 – 5.02]	3.43; [2.63 – 4.49]

Supplemental Table S9 (Supplement to figure 4)

Standardized (S-based) period seroprevalence at Baseline (BL), at FU1 (period between BL and FU1) and FU2 (period between FU1 and FU2). Shown are the population at risk in the general Tirschenreuth population and the analyzed cohort as absolute numbers as well as absolute numbers off all S-antibody positive, S&N positive and only S positive participants. Further shown is standardized and corrected seroprevalence (based on S-antibodies) (%) in the indicated groups with the 95% Wilson confidence intervals (95%-CI).

**Subgroup**	**population at risk** # County [# Cohort]	**S-antibody positive** # total [# S&N, # only S]	**Standardized total S positive** % [CI]	**Standardized S&N positive** % [CI]	**Standardized only S positive** % [CI]
**overall**	64379 [3371]	1503 [515, 988]	45.79 [44.11-47.47]	15.06 [13.89-16.31]	30.53 [29.00-32.10]
**sex**					
m	32102 [1559]	674 [242, 432]	44.48 [42.03-46.96]	15.29 [13.59-17.16]	28.96 [26.76-31.26]
w	32277 [1812]	829 [273, 556]	47.08 [44.79-49.39]	14.83 [13.27-16.54]	32.09 [29.98-34.28]
**age group**					
14 to 19	3994 [177]	51 [44, 7]	28.77 [22.60-35.83]	24.50 [18.75-31.33]	3.80 [1.83-7.73]
20 to 29	8145 [374]	129 [68, 61]	34.47 [29.84-39.43]	17.87 [14.32-22.07]	16.29 [12.90-20.38]
30 to 39	8430 [420]	135 [53, 82]	32.11 [27.83-36.72]	12.34 [9.53-15.83]	19.54 [16.03-23.60]
40 to 49	8781 [488]	210 [81, 129]	43.06 [38.74-47.49]	16.29 [13.28-19.83]	26.53 [22.81-30.62]
50 to 59	12802 [737]	296 [132, 164]	40.18 [36.70-43.76]	17.60 [15.02-20.51]	22.30 [19.45-25.45]
60 to 69	10389 [652]	263 [78, 185]	40.35 [36.65-44.16]	11.69 [9.44-14.38]	28.49 [25.16-32.08]
70 to 79	6712 [360]	274 [39, 235]	76.32 [71.66-80.41]	10.57 [7.80-14.17]	65.82 [60.77-70.53]
over 80	5126 [163]	145 [20, 125]	89.23 [83.54-93.12]	11.99 [7.86-17.87]	77.35 [70.34-83.11]
**senior care homes**					
without SCH	20730 [1114]	459 [155, 304]	42.25 [39.39-45.18]	13.49 [11.61-15.62]	28.58 [26.00-31.30]
with SCH	43649 [2257]	1044 [360, 684]	47.46 [45.41-49.53]	15.81 [14.36-17.37]	31.45 [29.57-33.40]
